# Structure, Dynamics and Cellular Insight Into Novel Substrates of the *Legionella pneumophila* Type II Secretion System

**DOI:** 10.3389/fmolb.2020.00112

**Published:** 2020-06-11

**Authors:** Theo J. Portlock, Jessica Y. Tyson, Sarath C. Dantu, Saima Rehman, Richard C. White, Ian E. McIntire, Lee Sewell, Katherine Richardson, Rosie Shaw, Alessandro Pandini, Nicholas P. Cianciotto, James A. Garnett

**Affiliations:** ^1^Centre for Host-Microbiome Interactions, Dental Institute, King’s College London, London, United Kingdom; ^2^Department of Chemistry and Biochemistry, School of Biological and Chemical Sciences, Queen Mary University of London, London, United Kingdom; ^3^Department of Microbiology and Immunology, Northwestern University Feinberg School of Medicine, Chicago, IL, United States; ^4^Department of Computer Science, Brunel University London, Uxbridge, United Kingdom

**Keywords:** *Legionella pneumophila*, effector, type II secretion system, structure, dynamics, NttA, NttC, NttE

## Abstract

*Legionella pneumophila* is a Gram-negative bacterium that is able to replicate within a broad range of aquatic protozoan hosts. *L. pneumophila* is also an opportunistic human pathogen that can infect macrophages and epithelia in the lung and lead to Legionnaires’ disease. The type II secretion system is a key virulence factor of *L. pneumophila* and is used to promote bacterial growth at low temperatures, regulate biofilm formation, modulate host responses to infection, facilitate bacterial penetration of mucin gels and is necessary for intracellular growth during the initial stages of infection. The *L. pneumophila* type II secretion system exports at least 25 substrates out of the bacterium and several of these, including NttA to NttG, contain unique amino acid sequences that are generally not observed outside of the *Legionella* genus. NttA, NttC, and NttD are required for infection of several amoebal species but it is unclear what influence other novel substrates have within their host. In this study, we show that NttE is required for optimal infection of *Acanthamoeba castellanii* and *Vermamoeba vermiformis* amoeba and is essential for the typical colony morphology of *L. pneumophila*. In addition, we report the atomic structures of NttA, NttC, and NttE and through a combined biophysical and biochemical hypothesis driven approach we propose novel functions for these substrates during infection. This work lays the foundation for future studies into the mechanistic understanding of novel type II substrate functions and how these relate to *L. pneumophila* ecology and disease.

## Introduction

*Legionella pneumophila* is a Gram-negative bacterium, which is ubiquitous in both natural and anthropogenic freshwater environments (Fliermans et al., 1981; Alary and Joly, 1991; Mouchtouri et al., 2007). *L. pneumophila* is highly prevalent in aquatic systems due to its ability to survive in biofilms and as an intracellular parasite of waterborne amoeba (Lau and Ashbolt, 2009; Taylor et al., 2009; Thomas et al., 2010). Replication of *L. pneumophila* is dependent on growth within a host and *L. pneumophila* can infect at least 20 amoeba species, including the genera *Acanthamoeba*, *Echinamoeba*, *Naegleria*, *Vahlkampfia, Vermamoeba*, and *Willaertia* (Rowbotham, 1980; Tyndall and Domingue, 1982; Anand et al., 1983; Holden et al., 1984; Newsome et al., 1985; Barbaree et al., 1986; Henke and Seidel, 1986; Wadowsky et al., 1991; Cianciotto and Fields, 1992; Fields, 1996; Michel et al., 1998; Molmeret et al., 2001; Miyamoto et al., 2003; Declerck et al., 2007; Dey et al., 2009; Harada et al., 2010; Buse and Ashbolt, 2011). Upon invasion of amoeba, *L. pneumophila* avoids fusion with host endosomal and lysosomal pathways by forming a modified phagosome, the *Legionella* containing vacuole (LCV) (Yong et al., 2010). *L. pneumophila* is an opportunistic human pathogen and as free-swimming bacteria or while encapsulated within protozoa, *L. pneumophila* can also enter human lungs via aerosolized droplets and infect local macrophages and epithelial cells (van Heijnsbergen et al., 2015). *L. pneumophila* is the major etiological agent of Legionnaires’ disease, an often-fatal pneumonia, and Pontiac fever, a milder flu-like disease (Pancer and Stypulkowska-Misiurewicz, 2003; Mercante and Winchell, 2015).

The Dot/Icm type IVb secretion system (T4SS) is a major determinant of *L. pneumophila* pathogenicity (Hubber and Roy, 2010; Schroeder, 2017). It transports >300 effector proteins directly into the host cytoplasm during infection, which are necessary for LCV development and intracellular replication. *L. pneumophila* also expresses a type II secretion system (T2SS) (White and Cianciotto, 2019). This is essential for both intra and extracellular survival with roles in biofilm formation, intracellular replication in amoeba and macrophages, dampening of cytokine output from infected cells, tissue and mucin degradation, and bacterial persistence in the lung (Rossier et al., 2004; DebRoy et al., 2006; McCoy-Simandle et al., 2011; White and Cianciotto, 2016, 2019; Mallama et al., 2017; White et al., 2018, 2019; Rehman et al., 2020). The overall structure of the *L. pneumophila* T2SS can be described in four parts: an outer membrane secretin pore that extends into the periplasm (LspD); an inner membrane platform (LspC, LspF, LspL, LspM); a cytosolic ATPase (LspE); and a pseudopilus (LspG, LspH, LspI, LspJ, LspK) (Gu et al., 2017; Ghosal et al., 2019). During export, type II substrates are recognized on their surface but must first enter the periplasm and fold into their native state, with the majority of substrates imported via the Sec pathway (Cianciotto and White, 2017). Although the mechanism of secretion is unclear, it is thought that interactions between substrates and the inner membrane platform, secretin and pseudopilus trigger transport into the extracellular environment, mediated by the pseudopilus and driven by ATP hydrolysis (Gu et al., 2017; Thomassin et al., 2017; Korotkov and Sandkvist, 2019).

The *Legionella* T2SS exports at least 25 proteins (DebRoy et al., 2006; White and Cianciotto, 2019). Many of these are upregulated during intracellular infection but their precise roles here are not clear, which may be due to functional redundancy between *Legionella* effectors. For example, the expression of *plaC* (acyltransferase), *lapA* and *lapB* (aminopeptidases) genes are significantly higher in *Legionella* during infection of *Acanthamoeba castellanii*, although neither single *plaC* (acyltransferase) or double *lapA*/*lapB* (aminopeptidases) mutants are impaired for infection (White et al., 2018). However, a double *lapA*/*plaC* mutant displays a ∼50-fold defect in infection, which implies that LapA and PlaC have complimentary roles in acquiring nutrients from the host. Functional redundancy enables *Legionella* to adjust to different host environments, which has a major impact on its broad host range and in turn contributes to large environmental reservoirs of *L. pneumophila* (O’Connor et al., 2011; Tyson et al., 2014). For example, the type II substrates NttA and NttD are required for optimal intracellular replication in *A. castellanii* but not human U937 macrophages or *Vermamoeba vermiformis* (NttA, NttD) or *Naegleria lovaniensis* (Tyson et al., 2013; White et al., 2018). Conversely, *nttC* mutants are impaired for infection of *V. vermiformis* and *Willaertia magna* but not *N. lovaniensis* or *A. castellanii* (Tyson et al., 2014), while *nttB* mutants show no impairment for infection of macrophages or several types of amoeba (Tyson et al., 2013).

NttE, like NttA, NttB, NttC, and NttD before it is considered one among numerous proteins secreted via the *Legionella* T2SS that contain “novel” amino acid sequences (White and Cianciotto, 2019). Some of these “novel” proteins share, to varying degrees, sequence similarity to hypothetical proteins in other bacteria, but more interestingly, others do not have any known homologues (*E*-value < 1 × 10^–10^) outside of the genus *Legionella* (DebRoy et al., 2006; Tyson et al., 2013, 2014; White et al., 2018; White and Cianciotto, 2019). Here, we demonstrate that expression of *nttE* is also required for optimal infection of *A. castellanii* and *H. vermiformis* amoeba and it is essential for the typical colony morphology of *L. pneumophila*. We also report the X-ray crystal structures of NttE and NttC, the solution nuclear magnetic resonance (NMR) structure of NttA and dynamic analyses for each substrate using complementary biophysical techniques. All three substrates form unique folds and through a combined cellular, biochemical/biophysical and bioinformatic approach, we propose putative functions for these type II substrates during *L. pneumophila* infection.

## Materials and Methods

### Bacterial Strains and Media

*L. pneumophila* strain 130b (American Type Culture Collection [ATCC] strain BAA-74) served as the wild-type strain in this study (Stewart et al., 2009). Legionellae were routinely grown at 37°C on buffered charcoal yeast extract (BCYE) agar, which, when appropriate, was supplemented with kanamycin at 25 μg/ml, or gentamicin at 2.5 μg/ml (Stewart et al., 2009). DNA was isolated from *L. pneumophila* strains as previously described (Cianciotto and Fields, 1992). *Escherichia coli* strain DH5α (Invitrogen, Carlsbad, CA, United States) was the host for recombinant plasmids. *E. coli* clones were grown in Luria-Bertani media with kanamycin (50 μg/ml), chloramphenicol (30 μg/ml), or ampicillin (100 μg/ml).

### Mutant Construction

In order to obtain *L. pneumophila* mutants lacking *nttE*, a fragment containing the 5′ end of the gene was amplified from strain 130b DNA using primers JS3 and SB34 (Integrated DNA Technologies; Coralville, IA, United States) (Supplementary Table 1), and a fragment containing the 3′ end of the gene was amplified using primers JS4 and SB35 (Supplementary Table 1). The two fragments were ligated into pGEM-T Easy (Promega, Madison, WI, United States), yielding pG02811a and pG02811b, respectively. Plasmids pG02811a and pG02811b were then digested with *Sma*I and *Spe*I, and a gentamicin-resistance cassette from pX1918-GT (Allard et al., 2006) was ligated in, to yield pG02811:Gt. Finally, pG02811:Gt was introduced into strain 130b by transformation (Chatfield et al., 2011), and mutant colonies were obtained on BCYE agar containing gentamicin. Verification of the *nttE* mutants was done by PCR, using primers JS3 and JS4. Two independent mutants derived in this way were designated as strains NU450 and NU451. A mutant of strain 130b containing a non-polar (unmarked) deletion in *nttE* was also constructed using a form of allelic exchange. To begin, mutagenized alleles were generated using overlap extension PCR (OE-PCR) as previously done (White and Cianciotto, 2016). The 5′ and 3′ regions flanking the open reading frame of *nttE* were PCR-amplified from 130b DNA with the use of primer pairs RW1/RW2 and RW3/RW4, respectively (Supplementary Table 1), and a kanamycin-resistance cassette flanked by Flp recombination target sites was similarly PCR-amplified from pKD4 by use of primer pair RW5/RW6 (Supplementary Table 1). Two-step OE-PCR was then done to combine the 5′ and 3′ regions of *nttE* with the resistance cassette. PCR products corresponding to the correct target size were gel purified and ligated into pGEM-T Easy to yield pGnttE:Kn. After transforming strain 130b with the newly made plasmid, bacteria containing an inactivated *nttE* were obtained by plating onto BCYE agar containing kanamycin and verified by PCR using primer pair RW1/RW4. Next, following electroporation (Cianciotto and Fields, 1992) of pBSFLP into the *nttE* mutant and subsequent plating onto BCYE agar containing 1 mM IPTG and gentamicin, the colonies obtained were patched onto ordinary BCYE agar in order to promote the loss of pBSFLP. Clones that were sensitive to gentamicin and kanamycin were isolated, and the loss of the plasmid and the chromosomal antibiotic cassette (leaving only an unmarked deletion) was confirmed by PCR. The new *nttE* deletion mutant was designated strain NU452. Complementation was not pursued due to the monocistronic nature of *nttE* and the common phenotypes subsequently displayed by all three independent *nttE* mutants.

### Assessments of Bacterial Extracellular Growth and Secreted Activities

*L. pneumophila* colony morphology was assessed after 7 days of incubation on BCYE agar. In order to further monitor the extracellular growth of *L. pneumophila* strains, legionellae grown on BCYE agar were inoculated into buffered yeast extract (BYE) broth and incubated at 37°C with shaking (Tyson et al., 2013). The optical density (OD) of each culture was then determined at 660 nm using a DU720 spectrophotometer (Beckman Coulter). Cell-free supernatants collected from late-log BYE cultures were assayed for protease activity as measured by azocasein hydrolysis, for phosphatase activity as measured by the release of *p*-nitrophenol from *p*-nitrophenol phosphate, and for lipase activity as measured by the release of *p*-nitrophenol from *p*-nitrophenol caprylate (Aragon et al., 2000, 2001, 2002).

### Intracellular Infection Assays

*Acanthamoeba castellanii* (ATCC 30234) and *V. vermiformis* (ATCC 50237) were axenically grown and maintained as previously described (Tyson et al., 2013). Amoeba were infected with *L. pneumophila* as done before (Rossier et al., 2008; Pearce and Cianciotto, 2009; Stewart et al., 2011; Tyson et al., 2013, 2014; White and Cianciotto, 2019). Briefly, stationary-phase bacteria were added, at a multiplicity of infection of 0.1, to 24-well tissue culture wells containing 1 × 10^5^ amoeba, and at various times post-inoculation, the numbers of legionellae in the co-culture were enumerated by plating aliquots on BCYE agar.

### Cloning, Expression and Purification

Full-length NttA (residues 1–101) and NttC (residues 1–108), minus their N-terminal periplasmic signal sequences, were amplified from the genomic DNA of *L. pneumophila* strain 130b using primer pairs JG21/22 and JG23/24, respectively, and cloned into the N-terminal His_6_-tagged vector pET-46 Ek/LIC (Novagen) (Supplementary Table 1). Expression of *nttA* and *nttC* was carried out in *E. coli* BL21 (DE3) cells (New England Biolabs). NttE (residues 1–269) from *L. pneumophila* strain 130b in pET-46 Ek/LIC was expressed as described previously (Rehman et al., 2020). Cells were grown in the presence of 50 μg/ml ampicillin at 37°C in either LB media (NttE), M9 minimal media supplemented with selenomethionine (Molecular Dimensions; NttC, NttE) or M9 minimal media supplemented with [U-^13^C_6_]glucose and/or ^15^NH_4_Cl (NttA). Expression was induced with 0.5 mM isopropyl-d-1-thiogalactopyranoside (IPTG) at an OD_600__nm_ of 0.6 and cells were harvested after growth overnight at 18°C. Cells containing NttA and NttE were resuspended in 20 mM Tris–HCl pH 8, 200 mM NaCl, 5 mM MgCl_2_, 1 mg/ml DNase I, 5 mg/ml lysozyme, lysed by sonication and purified using nickel affinity chromatography (Qiagen). Cells containing NttC were resuspended in 20 mM Tris–HCl pH 8, 8 M urea, lysed by sonication and purified using nickel affinity chromatography. NttC was then refolded by dialysis against 20 mM Tris–HCl pH 8, 1 M urea, 200 mM NaCl followed by 20 mM Tris–HCl pH 8, 200 mM NaCl. All samples were then gel filtered using a Superdex 75 (NttA, NttC) or 200 (NttE) column (GE Healthcare) equilibrated in 20 mM Tris–HCl pH 8, 200 mM NaCl.

### Crystal Structure Determination

NttC (15 mg/ml) and NttE (10 mg/ml) in 20 mM Tris–HCl pH 8, 200 mM NaCl were crystallized at 293K using the sitting-drop vapor-diffusion method grown in either 3.5 M ammonium chloride, 100 mM Tris–HCl pH 8.0 or 40% (v/v) ethylene glycol, 20% (w/v) polyethylene glycol 8000, 50 mM imidazole, 50 mM 2-(*N*-morpholine)ethanesulfonic acid pH 6.5, 300 mM magnesium chloride, 300 mM calcium chloride, respectively. Crystals were soaked in well solution plus additional 35% (w/v) xylitol (NttC) or 10% (v/v) polyethylene glycol 200 (NttE), flash cooled in liquid nitrogen and diffraction data were collected at 100K on beamline I04 at the diamond light source (DLS), United Kingdom. Data were processed with XDS and scaled with AIMLESS (NttC) or SCALA (NttE) using the XIA2 pipeline (Evans, 2006; Kabsch, 2010; Evans and Murshudov, 2013). The structure of NttC was determined with Se-MAD and NttE was determined with Se-SAD. Two sites were located in NttC and ten in NttE using SHELX (Sheldrick, 2008), and then phases were calculated using autoSHARP (Vonrhein et al., 2007). After automated model building with BUCCANEER (NttC) and ARP/wARP (NttE), the remaining structures were manually built within COOT (Cowtan, 2006; Langer et al., 2008). Refinement was carried out with REFMAC using non-crystallographic symmetry (NCS) and translation-libration-screw (TLS) groups, and 5% of the reflections were omitted for cross-validation (Murshudov et al., 2011). During refinement of NttC, refinement parameters were optimized using PDBredo (Joosten et al., 2009). Processing and refinement statistics of the final model can be found in Table 1.

**TABLE 1:** Crystal diffraction data and refinement statistics.

	**NttC**			**NttE**
	**Peak**	**Inflection**	**High remote**	**Peak**
**Crystal parameters**				
Space group	*P*6_2_22	*P*6_2_22	*P*6_2_22	*P*3_2_21
Cell dimensions (Å)	*a* = *b* = 84.39 *c* = 151.66	*a* = *b* = 84.39, *c* = 151.66	*a* = *b* = 84.39, *c* = 151.66	*a* = *b* = 120.87, *c* = 104.39
**Data collection**				
Beamline	DLS I04	DLS I04	DLS I04	DLS I04
Wavelength (Å)	0.9796	0.9797	0.9681	0.9793
Resolution (Å)	73.08–3.10 (3.18–3.10)	73.08–3.10 (3.18–3.10)	73.08–3.10 (3.18–3.10)	104.68–2.20 (2.26–2.20)
Unique observations	6280 (437)	6295 (439)	6295 (439)	45011 (3292)
R_merge_	0.283 (1.022)	0.297 (1.275)	0.356 (1.699)	0.071 (0.423)
R_pim_	0.064 (0.226)	0.067 (0.282)	0.080 (0.374)	0.024 (0.121)
<*I*>/*σI*	10.1 (3.7)	9.9 (3.1)	8.9 (2.5)	25.1 (7.2)
Completeness (%)	100 (100)	100 (100)	100 (100)	99.9 (99.5)
Redundancy	20.4 (20.9)	20.4 (21.2)	20.4 (21.4)	13.9 (14.0)
Wilson B-factor (Å^2^)	62.7			33.0
**Phasing**				
Figure of merit (accentric/centric)	0.348/0.337			0.441/0.097
Phasing power	0.771			1.964
**Refinement**				
R_work_/R_free_ (%)	25.0/28.9			18.7/21.0
Number of protein residues	216			526
Number of ligands				11 EDO
Average B-factor	64.0			42.0
**rmsd stereochemistry**				
Bond lengths (Å)	0.004			0.011
Bond angles (°)	1.281			1.698
**Ramachandran analysis**				
Residues in outlier regions (%)	0.0			0.0
Residues in favored regions (%)	98.1			97.5
Residues in allowed regions (%)	100			100

*Numbers in parentheses refer to the outermost resolution shell. *R*_sym_ = | *I* –<*I*>| /*I* where *I* is the integrated intensity of a given reflection and <*I*> is the mean intensity of multiple corresponding symmetry-related reflections. *R*_pim_ = (√1/n-1)| *I* –<*I*>| /*I* where *I* is the integrated intensity of a given reflection and <*I*> is the mean intensity of multiple corresponding symmetry-related reflections. *R*_work_ = | | F_o_| – | F_c_| | /F_o_ where F_o_ and F_c_ are the observed and calculated structure factors, respectively. *R*_free_ = *R*_work_ calculated using 10% random data excluded from the refinement. rmsd stereochemistry is the deviation from ideal values. Ramachandran analysis was carried out using Molprobity.*

### SAXS Data Collection and Analysis

Small angle X-ray scattering (SAXS) data were collected on beamline B21 at the DLS, United Kingdom at 25°C. 60 μl of NttE (10 mg/ml) in 20 mM Tris–HCl pH 8, 200 mM NaCl was applied to a KW403-4F column (Shodex) at 0.16 ml/min and SAXS data were measured over a momentum transfer range of 0.003 < *q* < 0.44 Å^–1^. Peak integration and buffer subtraction were performed in CHROMIXS (Panjkovich and Svergun, 2018). The radius of gyration (Rg) and scattering at zero angle [I(0)] were calculated from the analysis of the Guinier region by AUTORG (Franke et al., 2017). The distance distribution function [P(r)] was subsequently obtained using GNOM (Franke et al., 2017), yielding the maximum particle dimension (*D*_max_). The Porod volume (*V*_p_) was calculated with DATPOROD and molecular weight was estimated using MW = *V*_p_/1.7 (Franke et al., 2017). *Ab initio* low resolution shape restoration was performed using GASBOR (Svergun et al., 2001). Ten independent GASBOR runs were compared with SUPCOMB (Franke et al., 2017), and the model with the lowest normalized spatial discrepancy (NSD) score was chosen as a representative model. CRYSOL (Franke et al., 2017) was used to compare this model against the solution SAXS curve. Refinement of the full length NttE model was carried out with SASREF (Franke et al., 2017). Processing and refinement statistics can be found in Table 2.

**TABLE 2:** SAXS data and refinement statistics.

**SAXS data collection**	
**Beamline**	**DLS B21**
Wavelength (Å)	1.0
q Range (Å^–1^)	0.003 to 0.44
**Structural parameters**	
I(0)	0.0379 ± 5.7e-05
R_g_ (nm) (from Guinier)	2.94 ± 0.03
R_g_ (nm) (from P(r))	2.91 ± 0.05
D_max_ (nm) (from P(r))	9.40
Gasbor model χ^2^ fit	1.0
Gasbor models NSD	0.85
Gasbor/crystal structure NSD	2.87
Crystal structure χ^2^ fit	1.29
SASREF models χ^2^ fit	1.00, 1.19, 1.01, 1.08
**Molecular mass determination**	
MW (kDa) (from sequence)	33.2
MW (kDa) (from SAXS)	66.9

### Molecular Dynamics

Molecular Dynamics (MD) simulations were performed starting from the NttC X-ray structure (PDB ID code: 6SJT) using the GROMACS 2016 package (Abraham et al., 2015), with selenomethionine residues modified to methionine. The protein system was thermalized, equilibrated and simulated using an adaptation to the simulation protocol published by Fornili et al. (2013). From the final structure of pressure equilibration three independent production trajectories of 350 ns were generated. Structures were recorded every 2.5 ps for analysis. NttC trajectories were analyzed using tools in GROMACS 2016 package. Representative structures were extracted every 100 ps for pocket detection with fpocket (Le Guilloux et al., 2009).

### NMR Spectroscopy

NMR measurements were performed at 310 K on a ^15^N^13^C-labeled NttA sample in 50 mM NaPO_4_ pH 7.4, 50 mM NaCl, 10% D_2_O. NMR experiments for backbone and side-chain assignment were performed on two different Bruker spectrometers, an Avance III HD 700 and Avance III HD 950, equipped with TCI and TXI cryoprobes. Assignments were completed using standard triple-resonance assignment methodology and data were analyzed using ANALYSIS (Sattler et al., 1999; Vranken et al., 2005). A total of 91% of the potential backbone (e.g., disregarding the N-terminal methionine and proline residues) and 89% of the potential side-chain resonances were assigned; this corresponds to 97% and 95%, respectively, when the 15-residue N-terminal histidine tag is ignored. A single interleaved three-dimensional ^1^H-^15^N/^13^C NOESY-HSQC experiment (mixing time 120 ms at 950 MHz) provided the distance restraints used in the final structure calculation. {^1^H}-^15^N heteronuclear NOE, *T*_1_ and *T*_2_ relaxation times on a ^15^N -labeled NttA sample were also recorded at 800 MHz.

### NMR Structure Determination

The ARIA protocol for automated NOESY assignment interfaced with the CNS program was used for structure calculation and run on the NMRbox server (Rieping et al., 2007; Maciejewski et al., 2017). Secondary structure in the NttA domain was first identified using the chemical shift-based dihedral angle prediction software DANGLE (Vranken et al., 2005). For residues located in secondary structure, experimentally derived hydrogen bonds and Φ/φ backbone dihedral angles from DANGLE, were introduced as restraints in the ARIA structure calculation. A summary of NMR-derived restraints and statistics for the ten lowest energy structures after water refinement using a thin layer of explicit solvent is reported in Table 3.

**TABLE 3:** Summary of NMR structural restraints and statistics.

**Distance restraints**	
Total NOE	3063
Intraresidue	486
Sequential (| *i*-*j*|) = 1)	350
Short range (| *i*-*j*|) > 3)	268
Medium range (| *i*-*j*|) < 4)	84
Long range (| *i*-*j*|) > 5)	214
Ambiguous	1661
Dihedral angle restraints	184
*φ*	92
ψ	92
**Structural statistics^a^**	
**Violations**	
Distance restraints (Å)	0.017 ± 0.002
Dihedral angle restraints (°)	0.39 ± 0.061
**Deviation from idealized geometry**	
Bond length (Å)	0.0031 ± 0.000118
Bond angle (°)	0.467 ± 0.0134
Impropers (°)	1.153 ± 0.0414
**Average pairwise rmsd (Å)**	
Heavy atoms	0.57 ± 0.05
Backbone atoms	0.29 ± 0.07
**Structural quality^b^**	
**Ramachandran statistics**	
Favored regions (%)	89.5
Allowed regions (%)	95.6
Disallowed regions	5 residues^c^

*^*a*^Average values and standard deviations over the 10 lowest energy conformers with respect to the average structure. ^*b*^Percentage of residues in the Ramachandran plot regions determined by MOLPROBITY using an average structure from the 10 lowest energy conformers. ^*c*^All outliers are situated in highly dynamic regions.*

### Lipid Overlay Assay

Lipid strips (Echelon Biosciences) were blocked at room temperature for 1 h in TBST (50 mM Tris pH 7.5, 3 mM KCl, 137 mM NaCl, 0.1% Tween 20) containing 3% BSA and then incubated overnight with 300 pmol His-tagged NttA. The membrane was washed three times with blocking buffer and then incubated for 2 h at room temperature in the same buffer containing anti-His-HRP antibody (Sigma) diluted 1:2,000 and then treated with enhanced chemiluminescence substrate (ECL; Pierce) before detection by enhanced chemiluminescence.

### Phylogenetic Reconstruction and Sequence Alignment

Amino acid sequences with homology to mature *L. pneumophila* NttA, NttC and NttD from the 130b strain were identified using a slightly modified approach as described previously (White and Cianciotto, 2019). Briefly, homologs were identified by blastp using a minimum query coverage of 60%, and amino acid identity of 25%. *E*-value cut-offs were set to five to ensure that distantly related sequences were still identified and included in the subsequent analysis. Sequences were then aligned and phylogenetic trees were created in clustal omega (Madeira et al., 2019).

## Results

### NttE Is Required for Amoebal Infection

Previous proteomic analysis of culture supernatants from wild-type *L. pneumophila* 130b strain (a clinical isolate) and a T2SS (*lspF*) mutant identified NttE, a 288-amino acid protein encoded by the *lpw02811* ORF (*lpg0189* in strain Philadelphia-1; *lpp0250* in strain Paris; *lpl0249* in strain Lens), as a secreted substrate of the *L. pneumophila* T2SS (DebRoy et al., 2006). Recent analysis of the genome database indicated that NttE homologs are present within ∼65% of *Legionella* species and are absent from non-*Legionella* species (White and Cianciotto, 2019). Since that initial study, genome sequencing has revealed a NttE homology within the protozoan parasite *Aquicella siphonis* (Chen et al., 2019), compatible with the close evolutionary relationship between the *Legionella* and *Aquicella* genera (White and Cianciotto, 2019). Since three of the four previously characterized novel substrates of the *L. pneumophila* T2SS promote intracellular infection of an amoebal host(s) (Tyson et al., 2013, 2014; White et al., 2018), we posited that NttE might also be important for some aspects of infection. We therefore initiated the characterization of *L. pneumophila* mutants of strain 130b that were specifically inactivated for the *nttE* gene.

**FIGURE 1:**
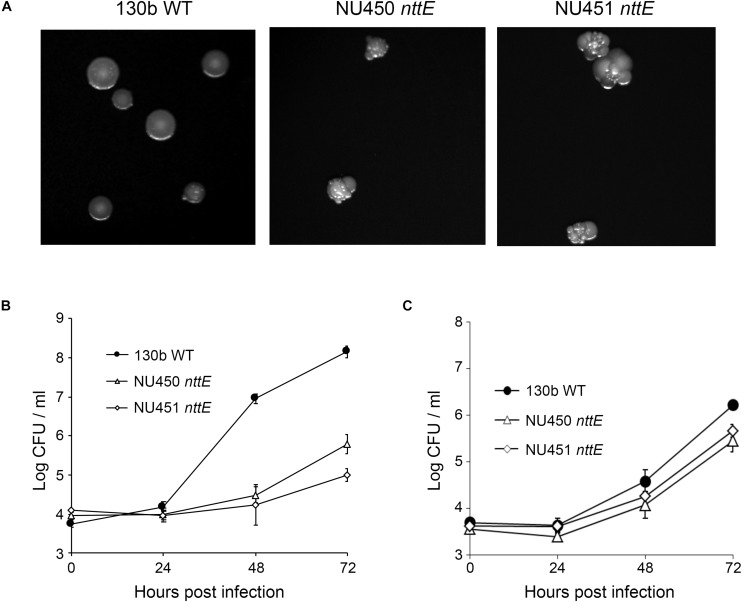
Colony formation of and intracellular infection of aquatic amoeba by *L. pneumophila* wild type and *nttE* mutant strains. **(A)** Wild-type (WT) 130b and *nttE* mutant strains NU450 and NU451 were plated onto standard BCYE agar and then colony morphology was observed after 7 days of growth at 37°C. **(B)** *A. castellanii* or (C) *V. vermiformis* amoeba were infected with either WT 130b or the *nttE* mutant NU450 or NU451 and at the indicated times, CFUs from the infected monolayers were determined. Data are the means and standard deviations from four infected wells. Data are representative of **(B)** three and **(C)** two independent experiments. For both amoebal hosts, the recovery of the mutants was significantly less than that of the WT at both 48 and 72 h (*P* < 0.05).

We initially generated two independently derived mutants, strains NU450 and NU451, that contained an antibiotic-resistance cassette within the *nttE* coding region. Mutant strain NU450 grew similarly to the parental wild-type in BYE broth (Supplementary Figure 1), indicating that the NttE protein is not required for optimal extracellular growth of *L. pneumophila*. Supernatants obtained from the mutant cultures contained wild-type levels of various known T2SS-dependent activities (Supplementary Figure 2), indicating that the loss of NttE also does not have a generalized effect on protein secretion. However, when cultured on BCYE agar plates, the *nttE* mutant exhibited an altered colony morphology which consisted of a more lobed pattern (Figure 1A). Even more significantly, the NU450 mutant was impaired for its ability to infect both *A. castellanii* and *V. vermiformis* amoeba (Figures 1B,C). Since the second independently derived *nttE* mutant, strain NU451, had the same mutant phenotypes as the first mutant (Figure 1), we inferred that the alterations in colony morphology and intracellular infection were due to the mutation in *nttE* and not a spontaneous, second-site mutation(s) in the chromosome. In addition, as *nttE* is monocistronic and not part of an operon and the genes upstream and downstream of *nttE* occur in the opposite orientation to that of *nttE*, we further inferred that the mutant phenotypes were due specifically to the lack of *nttE* (NttE) and not a dampening effect on an adjacent gene(s). In order to bolster this conclusion, we generated a third *nttE* mutant (strain NU452) which contained an unmarked (non-polar) deletion within the *nttE* coding region. The *nttE* deletion mutant behaved similarly to the two insertion mutants (Supplementary Figures 3, 4).

Taken together, these data indicate that NttE influences the colony morphology of *L. pneumophila*, possibly due to modification of a surface structure by secreted (extracellular) NttE and/or some surface-localization of the protein itself. Additionally, these data document that NttE is required for optimal intracellular infection of amoeba. However, given the novelty of NttE and the previously described *L. pneumophila* type II substrates NttA, NttC, and NttD, it has been difficult to predict based on primary sequence analysis what their functions are during infection. From our past and current cellular studies (Tyson et al., 2013, 2014; White et al., 2018), it is unclear what the precise role(s) of NttA, NttC, NttD, and NttE could be and we therefore employed structural and biophysical approaches with the hope that this would provide some level of functional insight. As the structure of NttD had previously been described (White et al., 2018), we proceeded to determine the structures of the remaining three substrates beginning with NttE.

### NttE Is a Dynamic Asymmetric Dimer in Solution

**FIGURE 2:**
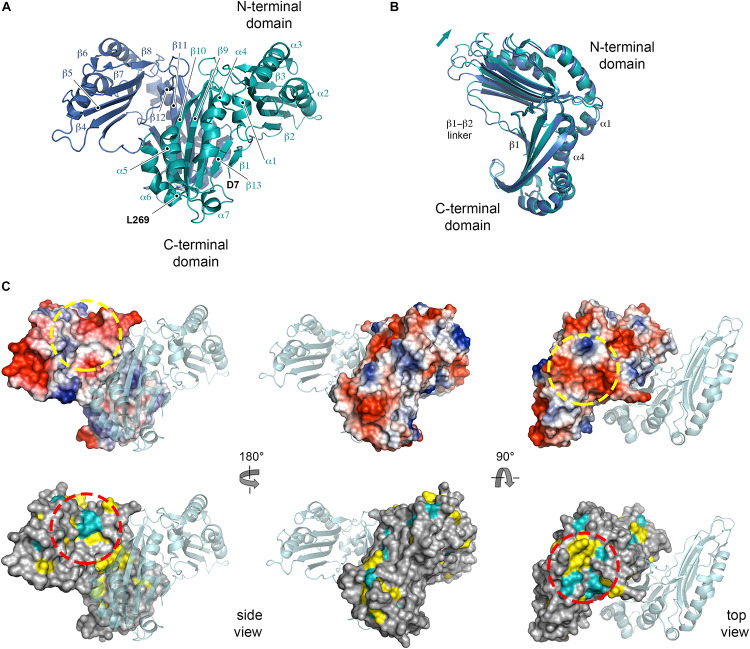
Crystal structure of NttE-130b. **(A)** Secondary structure of the NttE-130b dimer shown as cartoon. **(B)** Backbone superimposition of the C-terminal domains from the NttE-130b dimer highlighting the displacement of the N-terminal domain. Secondary structure elements at the NttE-130b inter-domain interface that propagate variations in the N-terminal domain orientation are annotated. **(C)** Electrostatic potential and sequence conservation of NttE shown on the NttE-130b monomer surface in three orientations. The other NttE-130b chain of the dimer is drawn as cartoon. The most prominent conserved patch on the NttE-130b surface is highlighted with a dashed red circle and the corresponding region on the electrostatic surface potential representation of NttE-130b is also highlighted with a dashed yellow circle.

Recombinant His_6_-tagged NttE (residues 1 to 269; minus its N-terminal signal sequences) was expressed in *E. coli* K12 strain and purified by nickel affinity. This was followed by size exclusion chromatography, which provided a molecular weight estimate of 78.9 kDa (theoretical mass 33.2 kDa) and suggested that NttE is either a dimer or trimer in solution (Supplementary Figure 5). Crystals of NttE were obtained at pH 6.5 and the structure was determined using selenium single-wavelength anomalous dispersion (Se-SAD) phasing with electron density maps refined to 2.2 Å (Table 1). The final model contains two molecules in the asymmetric unit and all residues could be built except for the N-terminal His_6_ tags and the NttE residues Asn1 to Ala6 (Figure 2A). Each NttE protomer is formed of two domains, with the N-terminal domain (residues T20 to N143) composed of 7 α-helices and 3 β-strands, and the C-terminal domain (residues D7 to F11 and G146 to L269) formed from 5 α-helices and 4 β-strands. Each domain is separated by a short linker and in the C-terminal domain the β1 strand at the N-terminus folds back against the β13 strand. Two disulfide bonds are present in each chain of NttE, one in the N-terminal domain between Cys62 and Cys90, and another in the C-terminal domain between Cys259 and Cys268. The NttE dimer is mainly stabilized through β-sheet interactions provided by the C-terminal domain and the presence of this dimer in the asymmetric unit strongly indicates that NttE is a dimer in solution.

During the preparation of this manuscript two alternative structures of NttE from the *L. pneumophila* Philadelphia 1 strain (NttE-Phil) have been described (PDB ID code 6L6G and 6L6H), which are derived from native and selenomethionine labeled proteins (Chen et al., 2019). NttE-Phil shares 99% sequence identity with NttE from the 130b strain (herein called NttE-130b) and both structures also exist as a dimer. However, while both selenomethionine labeled NttE-Phil and NttE-130b share a highly similar conformation, native NttE-Phil displays significant deviations in the position of its N-terminal domains (RMSD over all C_α_ atoms of 0.31 and 0.94 Å, respectively) (Supplementary Figure 6). Further analysis of each NttE-130b chain revealed similar deviations. Whilst the C-terminal domains are highly similar to one another (RMSD of 0.18 Å over all C_α_ atoms), the N-terminal domains display structural variation with a shift in the position of the α1 helix (RMSD of 0.31 Å over all C_α_ atoms) (Supplementary Figure 7). The NttE α1 helix mediates hydrophobic interactions with the α4 helix in the C-terminal domain, and it appears that small changes in this interface results in large changes in the orientation of the N-terminal region (Figure 2B).

**FIGURE 3:**
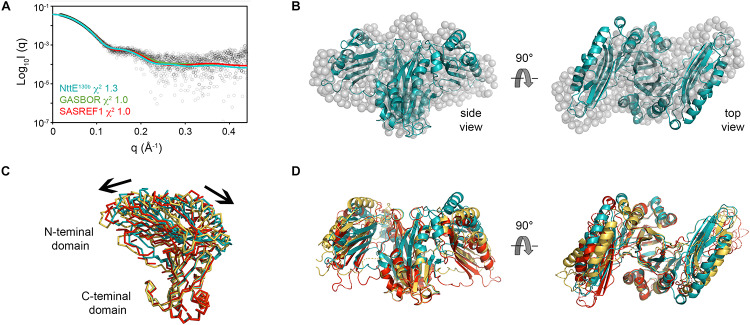
SAXS analysis of NttE-130b. **(A)** Fit of the crystal structures of NttE-130b (teal line) and GASBOR bead model (green line), and SAREF refined NttE-130b (red line) to the NttE-130b SAXS data (black open circles) with χ^2^ of 1.3, 1.0, and 1.0, respectively. **(B)** Superimposition of the NttE-130b crystal structure (teal cartoon) onto the GASBOR bead model of NttE-130b (gray spheres), shown in two orientations. **(C)** Backbone superimposition of the C-terminal domains from the NttE-130b crystal structure (teal) and SASREF (orange and red) highlighting significant displacement of the N-terminal domains between monomer (ribbons) and **(D)** within the dimer (cartoon) of the SAXS refined structures.

Examination of NttE-130b using the DALI server (Holm and Rosenstrom, 2010) revealed no other structures with significant tertiary homology. However, analysis of all known sequences of NttE homologs from *Legionella* species and *A. siphonis* revealed a conservation hotspot at the interface of the N- and C-terminal domains, positioned on the “top” face of the NttE dimer (Figure 2C, Supplementary Figure 8). This implied that NttE might bind a single ligand at each inter-domain site or a single oligomeric/polymeric molecule simultaneously at both. As we had observed structural variation in the NttE-Phil and NttE-130b crystal structures in this region, we hypothesized that NttE may use a conformational selection mechanism to bind this putative ligand. We therefore used size exclusion chromatography coupled to small angle X-ray scattering (SEC-SAXS) to evaluate the dynamics of NttE-130b in solution (Table 2). Guinier analysis suggested a radius of gyration (R_g_), the root mean square distance to the particles centre of mass, of 2.94 nm and analysis of the distance distribution function [P(r)] suggested a maximum particle dimension (*D*_max_) of 9.40 nm and R_g_ of 2.91 nm (Supplementary Figure 9). Evaluation of the Porod volume (114 nm^3^) provided a molecular mass of 66.9 kDa, which is consistent with a dimeric 66.4 kDa NttE-130b. Kratky plot analyses of the SAXS data indicated that NttE-130b displays some dynamic features in solution (Supplementary Figure 9) and evaluation of the calculated solution scattering from the NttE-130b structure provided a χ^2^ value of 1.3 (Figure 3A).

We next initiated *ab initio* dummy residue reconstructions of NttE-130b, assuming P2 symmetry, which yielded reproducible models with an average normalized spatial discrepancy (NSD) score between reconstructions of 0.9 and a χ^2^ fit between calculated and experimental solution scattering of 1.0 (Figures 3A,B). However, superposition of the NttE-130b crystal structure onto the dummy residue model provided a poor NSD score of 2.9, which reflects a deviation in some regions of the overall fit (Figure 3B). We therefore refined the orientation of the NttE-130b domains against the SAXS data using the program SASREF and this resulted in four new NttE-130b models with χ^2^ values against the experimental data of 1.0, 1.2, 1.0, and 1.1. Comparison of the two models with lowest χ^2^ values with the crystal structure of NttE-130b highlighted significant variability in the positioning of the N-terminal domains, although in all models the β1 strand remained tethered to the C-terminal domain (Figures 3C,D). Together these data indicate that the N-terminal domains of NttE are mobile and this may be important for its function in modifying the bacterial cell surface and/or during intracellular infection.

### NttC Contains a Breathable Internal Cavity

**FIGURE 4:**
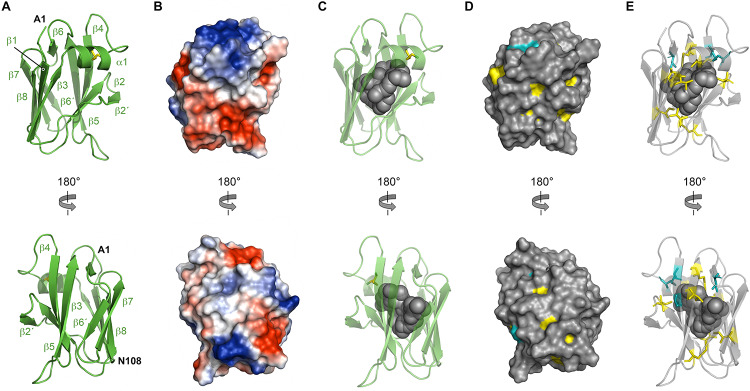
Crystal structure of NttC. **(A)** Secondary structure of NttC shown as cartoon in two orientations. **(B)** Electrostatic potential of the NttC surface. **(C)** NttC shown as cartoon with the internal cavity (calculated by fpocket) shown as spheres. **(D)** Sequence conservation of NttC shown on the surface structure and **(E)** as sticks with the internal cavity shown as spheres. Amino acid identities and similar residues are indicated by background shading in cyan and yellow, respectively.

NttC is a 130-amino acid protein encoded by the *lpw18401* ORF in 130b strain (*lpg1809* in strain Philadelphia-1; *lpp1772* in strain Paris; *lpl1773* in strain Lens) (DebRoy et al., 2006). It is present in ∼85% of *Legionella* species but has no detectable sequence homology outside of the *Legionella* genus (White and Cianciotto, 2019). As NttC has a key role during *L. pneumophila* infection of *V. vermiformis* and *W. magna* amoeba (Tyson et al., 2014) and is present in a large number of *Legionella* strains, we next turned our attention to determining its tertiary structure. Recombinant His_6_-tagged NttC (residues 1 to 108; minus its N-terminal signal sequences) was expressed in *E. coli* K12 strain but was insoluble and formed inclusion bodies. NttC was therefore purified using nickel affinity chromatography under denaturing conditions and refolded using dialysis through slow removal of urea. This was followed by size exclusion chromatography, which suggested a mass of 9.7 kDa (theoretical mass 13.4 kDa) and indicated that NttC is monomeric in solution (Supplementary Figure 5). We readily obtained crystals of NttC at pH 8.0 and determined its structure using selenium multi-wavelength anomalous dispersion (Se-MAD) and refined electron density maps to 3.1 Å (Table 1). The final model of NttC contains two identical molecules in the asymmetric unit (RMSD over C_α_ atoms of 0.080 Å) and all residues could be built except for the N-terminal His_6_ tags. NttC has an immunoglobulin-like fold composed of one α-helix and ten β-strands, and residues Cys46 and Cys55 form a disulfide bond between the α1-helix and β5-strand (Figure 4A, Supplementary Figure 10).

Examination of the charge distribution in NttC showed residues from the β5 and β6 strands create a hydrophobic patch on the NttC surface, while the remaining surface is composed of both positive and negative regions (Figure 4B). However, NttC is unusual in that it lacks a compact core and instead contains a buried internal cavity (Figure 4C). This is formed primarily by the sidechains of hydrophobic residues and is inaccessible to the solvent. Analysis of the structure using fpocket (Le Guilloux et al., 2009) revealed that the cavity inside the protein has a volume of 782 Å^3^ and a druggability score (Schmidtke and Barril, 2010) of 0.97, which strongly suggests that this pocket may be able to bind one or more organic molecules. Analysis of the NttC structure using the DALI server (Holm and Rosenstrom, 2010) identified a single structure, the C-terminal domain of the *Dictyostelium discoideum* Ca^2+^-dependent cell adhesion molecule (DdCAD-1) (PDB ID code 1B1O; Z score 7.0; rmsd 3.4), as having tertiary homology (Z score cut off 6.8) (Holm et al., 2008; Supplementary Figure 11). DdCAD-1 binds Ca^2+^ in its N-terminal domain and within its N-/C-terminal domain interface but these metal binding residues are not conserved in NttC. The role of the DdCAD-1 C-terminal domain is to promote dimerization and adhere DdCAD-1 to cell surfaces, and although protein:protein binding could be a shared property with NttC, DdCAD-1 lacks an internal cavity. Furthermore, when we analyzed all known sequences of NttC homologs (Supplementary Figures 12, 13) and mapped the sequence conservation onto the NttC structure, we observed only minor patches on the NttC surface while many of the residues that form the internal cavity were highly conserved (Figures 4D,E).

**FIGURE 5:**
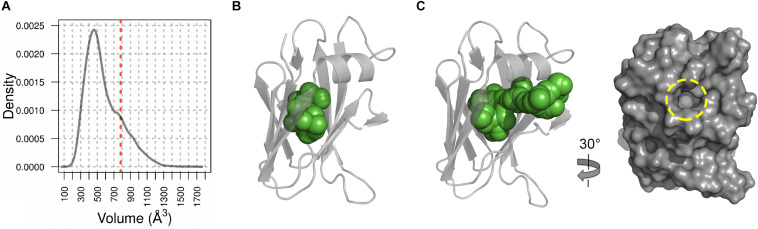
Molecular Dynamics analysis of NttC. **(A)** Distribution of the cavity volume from the NttC MD ensemble, with druggability score ≥0.8. Volume of the cavity from X-ray structure with draggability score of 0.97 is shown as red dashed line. **(B)** and **(C)** NttC snapshots from MD ensemble with pocket volumes 399 Å^3^ and 1106 Å^3^, respectively. NttC is shown as cartoon with pockets cavity (calculated by fpocket) shown as spheres. The latter structure is also rotated and shown as a surface representation to highlight the solvent accessible channel (yellow dashed circle).

We therefore hypothesized that this cavity forms a binding site for a yet to be identified cofactor or ligand and so we probed this further using Molecular Dynamic (MD) simulations. Based on root mean square fluctuation (RMSF) analysis of the NttC MD ensemble, 50% of the protein residues had an RMSF smaller than the median (0.7 Å) while 42% of residues, excluding the termini, had higher flexibility than the median (Supplementary Figure 14). When we mapped these regions of higher flexibility onto the crystal structure, they localized to the NttC poles within the α1 helix, the β2′, β3 and β6 strands and the adjacent loops. Using fpocket, we also analyzed the cavities in structures extracted from the NttC MD ensemble. 46% of the analyzed structures had cavities with a druggability score ≥0.8 (Figure 5A), and with volumes ranging from 177 to 1658 Å^3^ and a median volume of 521 Å^3^ (Figures 5B,C). Furthermore, in the ensemble structures with the largest cavities, a channel was often formed with an opening of 12 Å at the mouth, between the residues Ser22 and Gly48 (Figures 5B,C). Together these data suggest that the NttC cavity is dynamic, can allow entry to small molecules and has the potential to bind a variety of organic molecules.

### NttA Forms a Helical Bundle Structure

NttA is a 125-amino acid protein encoded by the *lpw13951* ORF (*lpg1385* in strain Philadelphia-1; *lpp1340* in strain Paris; *lpl1336* in strain Lens) (DebRoy et al., 2006). Although NttA is required for optimal infection of *A. castellanii* and *W. magna* (Tyson et al., 2013, 2014), its function remains unknown and so we finally focused on this substrate. Recombinant His_6_-tagged NttA (residues 1 to 101; minus its N-terminal signal sequences) was expressed in *E. coli* K12 strain and purified by nickel affinity, followed by size exclusion chromatography where NttA eluted as a minor and major species. Comparison of the NttA elution volumes against standard globular proteins provided a molecular mass of 21.9 kDa and 11.9 kDa (theoretical mass 13.3 kDa), respectively, which suggested that NttA is primarily monomeric in solution but has some propensity to form dimers (Supplementary Figure 5). Attempts to crystalize NttA were unsuccessful and so we instead elucidated the structure of monomeric NttA using solution heteronuclear NMR methods (Supplementary Figure S15).

**FIGURE 6:**
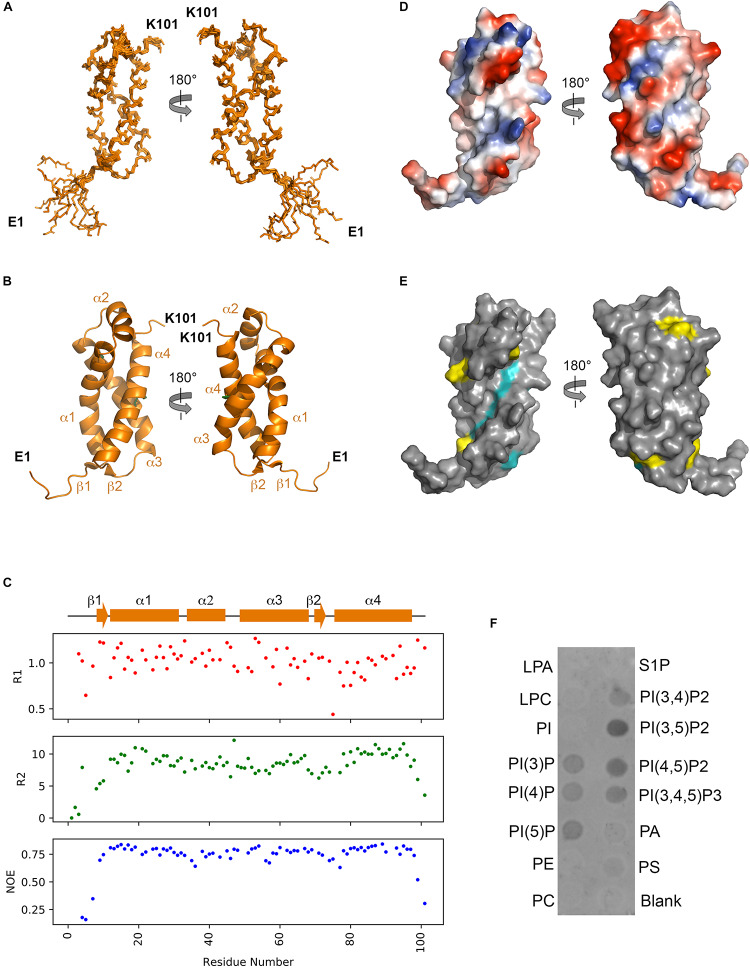
Solution structure of NttA. **(A)** Backbone superimposition of the ten best NMR structures of NttA shown in two orientations. **(B)** Secondary structure of one of the final NttA structures shown as cartoon. **(C)** T1, T2 and {^1^H}-^15^N heteronuclear NOE cross-relaxation rates for residues with well-resolved peaks in ^1^H^15^N NMR spectra. Those that were not well resolved are shown as blank spaces. Secondary structure elements are displayed above. **(D)** Electrostatic surface potential of NttA. **(E)** Sequence conservation of NttA shown on the surface structure. Amino acid identities and similar residues are indicated by background shading in cyan and yellow, respectively. **(F)** Detection of NttA binding to cellular lipids by protein-lipid overlay assay, using a rabbit anti-His antibody. LPA: lysophosphatidic acid; LPC: lysophosphocholine; PI: phosphatidylinositol; PI(3)P: phosphatidylinositol-3-phosphate; PI(4)P: phosphatidylinositol-4-phosphate; PI(5)P: phosphatidylinositol-5-phosphate; PE: phosphatidylethanolamine; PC: phosphatidylcholine; S1P: sphingosine-1-phosphate; PI(3,4)P2: phosphatidylinositol-3,4-bisphosphate; PI(3,5)P2: phosphatidylinositol-3,5-bisphosphate; PI(4,5)P2: phosphatidylinositol-4,5-bisphosphate; PI(3,4,5)P3: phosphatidylinositol-3,4,5-triphosphate; PA: phosphatidic acid; PS: phosphatidylserine. Blots are representative of two independent experiments.

From both manual and ARIA (Ambiguous Restraints for Iterative Assignment) NMR assignment methods, a total of 3063 nuclear Overhauser effects (NOEs) were assigned in NttA ^15^N/^13^C-edited NOESY spectra at pH 7.4, and structure determination was also supplemented with Φ/φ dihedral angles. The average pair-wise root-mean squared deviation (RMSD) for the water-refined final structures is 0.29 ± 0.05 Å for the backbone atoms and 0.57 ± 0.05 Å for the heavy atoms of residues within secondary structure. Structural statistics are shown in Table 3. NttA is composed of a three-helix bundle with an additional short helix (α2) bridging the α1 and α3 helices at the C-terminal face (Figures 6A,B, Supplementary Figure 16). A short β1-β2 sheet is also present on the N-terminal face along with two disulfide bonds between the α1-α3 (Cys27-Cys50) and α3-α4 (Cys61-Cys88) helices. All areas of secondary structure are well defined, however, there is increased flexibility at the N- and C-terminus supported by measurement of R1, R2 and {^1^H}-^15^N heteronuclear NOE parameters, which report on ns-ps timescale motions (Figure 6C).

The *nttA* gene is present in ∼75% of *Legionella* species (White and Cianciotto, 2019) but we also identified homology outside of the *Legionella* genus in the Gram negative bacteria *Rhizobiales bacterium*, *Deltaproteobacteria bacterium* and *Wenzhouxiangellaceae bacterium*. Interestingly, we also observed an additional ∼100 amino acid extension at the N-terminus of NttA in several *Legionella* species, namely *Legionella lansingensis*, *Legionella brunensis*, *Legionella jamestowniensis*, *Legionella hackeliae* and *Legionella jordanis* (Supplementary Figures 17, 18). When we compared this region with NttA from *L. pneumophila* 130b, there was clear sequence conservation, which included the four disulfide forming cysteine positions (Supplementary Figure 19). We concluded that in these strains NttA is present as a gene duplication connected by a flexible linker (corresponding to the unstructured N-terminal region in *L. pneumophila*), although it was unclear whether this has any functional importance. We next analyzed the electrostatic surface of NttA and observed that the α1-α3 helical face is predominantly negatively charged, whilst a noticeable hydrophobic cavity exists between the α1 and α4 helices on the opposite side (Figure 6D). Further evaluation of the sequence conservation of NttA highlighted two regions of potential functional importance (Figure 6E, Supplementary Figures 17, 19): the α2-α3 loop and the α1-α4 hydrophobic cavity.

Using the DALI server (Holm and Rosenstrom, 2010) we identified the *E. coli* RNA polymerase sigma factor RpoD (PDB ID code 4LK1; Z score 6.2; rmsd: 2.7), the *Streptomyces viridosporus* prenyltransferase MoeN5 (PDB ID code 5B0L; Z score 6.4; rmsd 2.8) (Zhang et al., 2016) and the *Solanum tuberosum* Ran GTPase-activating protein RanGAP2 (PDB ID code 4M70; Z score 6.6; rmsd 3.5) (Hao et al., 2013) as having tertiary homology with NttA (Z score cut off 6.1) (Holm et al., 2008; Supplementary Figure 20). MoeN5 belongs to the prenylsynthase family of enzymes and interacts with its ligands via a DDxD motif. NttA overlays well with the MoeN5 N-terminal region and although it lacks this conserved sequence, two acidic residues (Asp92 and Glu95) are located within close proximity. Prenylsynthase family enzymes are considered two domain proteins and as NttA has propensity to form dimers and is expressed as a tandem repeat protein in some *Legionella* species, it is tempting to speculate that NttA is functional as a dimer. NttA also overlays well with RpoD sigma factor in complex with RNA polymerase. Here the NttA α1 and α3 helices are positioned on the interface and NttA would presumably function as a competitor of RpoD, but little NttA sequence conservation is observed on this face of NttA. Finally, through tertiary homology with RanGAP2, NttA could also bind a Rx-Ran GTPase-like protein through its α1 and α4, yet no sequence homology for *S. tuberosum* Rx Ran GTPase is observed in *A. castellanii* or *W. magna*. So, based on tertiary structure analysis, it remained unclear what the role of NttA could be during infection.

### NttA Interacts With Phosphoinositides

At least 5% of effectors secreted by the *L. pneumophila* T4SS contain domains that can mediate binding to and/or processing of phosphoinositides present on the surface of the LCV (Hsu et al., 2012; Nachmias et al., 2019). For example, SidC contains a four-helix bundle (P4C) domain that binds phosphatidylinositol-4-phosphate (PI4P) in mature LCVs and then facilitates the recruitment of host endoplasmic reticulum proteins to the vacuole surface (Luo et al., 2015). As NttA also contains high helical content, we pondered whether it too could interact with host lipids and so we assayed its binding to phosphoinositides and other lipids immobilized onto a nitrocellulose membrane (Figure 6E). Surprisingly, under these conditions NttA bound to all phosphorylated forms of phosphoinositides but displayed a clear preference for phosphatidylinositol-3,5-biphosphate [PtdIns(3,5)P_2_] and possibly phosphatidylinositol-4,5-biphosphate [PtdIns(4,5)P_2_]. NttA showed no binding to phosphatidylinositol, phospholipids or lysosphingolipids, and these results indicate that NttA is a phosphoinositide binding protein and may be targeted to host organelles, such as the LCV, during intracellular infection.

## Discussion

In this study, we have shown that NttE is required for optimal intracellular infection of amoeba. Based upon the magnitude of the observed mutant defects, it appears that NttE is more important for the infection of *A. castellanii* than it is for the infection of *V. vermiformis*. Such a result is entirely in-line with other T2SS-dependent proteins that have proved to be more important in one amoebal host versus another amoebal host (Tyson et al., 2013, 2014; White et al., 2018). We previously characterized *L. pneumophila* mutants that lack a functional T2SS and showed that type II substrates are not required for *L. pneumophila* entry into macrophages or early evasion of the lysosomal degradation pathway (White and Cianciotto, 2016, 2019). However, the T2SS is necessary for correct Rab1B binding to LCVs and for intravacuolar growth of *L. pneumophila* during the initial phase post-infection (White and Cianciotto, 2016; White et al., 2019). Therefore, NttE is likely promoting intravacuolar replication; and joining NttA, NttC, and NttD (Tyson et al., 2013, 2014; White et al., 2018), NttE is now the fourth “novel” T2SS substrate demonstrated to be required for optimal intracellular infection.

We have also shown that NttE influences the colony morphology of *L. pneumophila* and this is possibly due to modification of a *L. pneumophila* surface structure by secreted (extracellular) NttE and/or surface-localization of the protein itself. Through our biophysical characterization of NttE, it is clear that this substrate is functional as a dimer and it displays significant inter-domain flexibility. We have presented a potential binding site for NttE, localized to the inter-domain surfaces, however, it is not clear whether this represents a site for a functional ligand or a targeting interface for potential association of NttE with the *L. pneumophila* surface. There is precedent for some *L. pneumophila* T2SS substrates to localize to the bacterial surface. For example, Lcl is a substrate involved in the initiation of early biofilm formation (Mallegol et al., 2012), while ChiA can degrade components of the complement system and facilitate bacterial penetration of host mucins (Rehman et al., 2020). Interestingly, although targeted to the bacterial surface (Rehman et al., 2020), ChiA is also fully secreted into the extracellular space and, during *L. pneumophila* infection of a human macrophage cell line, can escape the LCV and associate with the cytoplasmic face of the LCV (Truchan et al., 2017). However, it is still not clear what the role of NttE is during intracellular bacterial growth and whether it is trafficked into the host cytoplasm or whether it is retained within the lumen of the replication vacuole.

We previously demonstrated that NttC is necessary for optimal *L. pneumophila* infection of *V. vermiformis* and *W. magna* (Tyson et al., 2014) and we have now determined that NttC is a small monomeric protein with an unusual internal cavity. Due to the restricted access to this pocket we expect that a large cofactor is inserted during the folding of NttC, prior to its secretion from the periplasm. However, it is unusual that a co-factor would be completely buried from the solvent. In our crystal structure, we could not detect any access to the cavity but using MD we were able to observe a breathable core and a transient ∼12 Å diameter solvent accessible channel form over the course of the simulations. A similar size channel has been observed in the active site of triosephosphate isomerase (Wade et al., 1993) and this suggests that the opening in NttC would be able to accommodate ligands as large as dihydroxyacetone phosphate and D-glyceraldehyde-3-phosphate. It seems likely that NttC has an enzymatic function which is carried out in the core of its structure, and although it is difficult to speculate what the exact function might be, a NttC product could provide nutrition or affect host signaling. Alternatively, NttC may bind a small molecule that when released can modulate host immunity. For example, insect nitrophorins bind nitric oxide, which is released in their host during feeding to induce vasodilation (Knipp and He, 2011). The *nttC* gene has been detected in the majority of *Legionella* species (White and Cianciotto, 2019) and this indicates that NttC has a fundamental role during intracellular growth of *L. pneumophila*. Identifying its cofactor will now be essential to understand how it is able to promote infection.

Along with the crystal structures of NttE and NttC, in this study, we determined the solution structure of NttA, which has a unique helix bundle fold and is stabilized by two inter-helical disulfide bonds. A striking feature of NttA is a substantial hydrophobic groove that runs along its α1-α4 helix interface but based on tertiary structure analysis it is still not clear what function NttA may have during infection. However, we have also shown that NttA displays broad specificity for phosphorylated phosphoinositides. Phosphoinositides are eukaryotic lipids that have a major role in the regulation of cell signaling pathways and membrane trafficking. As such, *L. pneumophila* secretes via its T4SS a plethora of effectors that target host phosphoinositide lipids and help to establish and maintain the LCV (Weber et al., 2006; Hilbi et al., 2011). Here these effectors either manipulate phosphoinositide lipid chemistry or use them as anchors to modulate the trafficking of host organelles to the cytoplasmic face of the LCV or other host membranes. Although this could be due to limitations associated with immobilizing lipids, we do see evidence that NttA preferentially binds to PtdIns(3,5)P_2_ and PtdIns(4,5)P_2_. PtdIns(4,5)P_2_ is located in the plasma membrane from which the LCV is initially derived (Weber et al., 2014), whereas PtdIns(3,5)P_2_ is observed in early endosomes (McCartney et al., 2014). However, as the LCV matures it becomes enriched in phosphatidylinositol-4-phosphate (PtdIns4P), through the function of secreted *L. pneumophila* phosphatase and kinase effectors (Weber et al., 2006). This suggests that NttA is transported into the host cytoplasm and can associate with these lipids within the LCV, and that NttA is likely active during the earlier stages of intracellular infection. While the precise function of NttA remains unknown, potential localization suggests that NttA could either modify phosphoinositides or use these lipids as an anchor to carry out another function (e.g., interactions with host Rab or ubiquitination pathways) where association with the LCV is essential.

It is strongly believed that during translocation out of the bacterium, substrates of type II secretion systems are recognized through a three-dimensional recognition motif present on their surface (Gu et al., 2017; Thomassin et al., 2017; Korotkov and Sandkvist, 2019). However, the nature of this motif and how it is sampled by different type II secretion systems remains unclear. In *L. pneumophila*, the T2SS exports an unusually large number of substrates and with contributions from this study this represents the largest catalog of intact and subdomain substrate structures from any one T2SS; namely LapA, LapB, Map, NttA, NttB, NttC, NttD, NttE, NttG, and the ChiA C-terminal chitinase/mucinase domain (Dhatwalia et al., 2015; Zhang et al., 2017a, b; Gong et al., 2018; White et al., 2018; Chen et al., 2020; Rehman et al., 2020). Furthermore, the *L. pneumophila* T2SS appears to transport two of the smallest substrates that have identified to date, NttA and NttC (11.5 kDa each), although association of a co-factor with NttC could promote its oligomerization. The *L. pneumophila* T2SS is therefore emerging as a model system to not just understand the biology of type II dependent intracellular bacterial growth but also more generally how substrates are recognized and exported by type II secretion systems.

In summary, using a combined cellular, structural, biophysical and biochemical approach we have provided a characterization of three novel type II secreted substrates, NttA, NttC and NttE, that are required for infection of several species of amoeba. Amoeba play a key role in the transmission of *L. pneumophila* from aquatic habitats to the human host; therefore, the finding that these T2SS substrates promote amoebal infection is relevant to human disease. Nonetheless, it will be important for future studies to investigate the role on these novel proteins in *L. pneumophila* infection of human macrophages and the mammalian lung. This study offers clues for the function of these fascinating substrates during infection and now further work is needed to provide mechanistic insight.

## Data Availability Statement

The assigned chemical shifts of NttA have been deposited in the BioMagResBank (http://www.bmrb.wisc.edu/) with accession code 34480. Coordinates for NttA, NttC, and NttE have been deposited in the PDB (https://www.rcsb.org/) with accession codes 6XTT, 6SJT, and 6SKW, respectively. The buffer subtracted NttE SAXS curve and GASBOR model with the lowest NSD score have been deposited in the Small Angle Scattering Biological Data Bank (https://www.sasbdb.org) with accession ID SASDHW2.

## Author Contributions

TP, JT, SD, SR, RW, IM, AP, NC, and JG conceived and designed the experiments. TP, JT, SD, SR, RW, IM, LS, KR, RS, and JG performed the experiments. TP, JT, SD, SR, RW, IM, LS, AP, NC, and JG analyzed the data. AP, NC, and JG wrote the manuscript and contributed reagents, materials, and analysis tools.

## Conflict of Interest

The authors declare that the research was conducted in the absence of any commercial or financial relationships that could be construed as a potential conflict of interest.
