# Supplementary material for: Structure, Dynamics and Cellular Insight Into Novel Substrates of the Legionella pneumophila Type II Secretion System
Source: Front Mol Biosci. 2020 Jun 11;7:112. doi: 10.3389/fmolb.2020.00112 (PMC7325957; doi:10.3389/fmolb.2020.00112)
Supplement: Supplementary file 1 [file Data_Sheet_1.PDF]

Supplementary Material

1 Supplementary Figures and Tables

1.1 Supplementary Figures

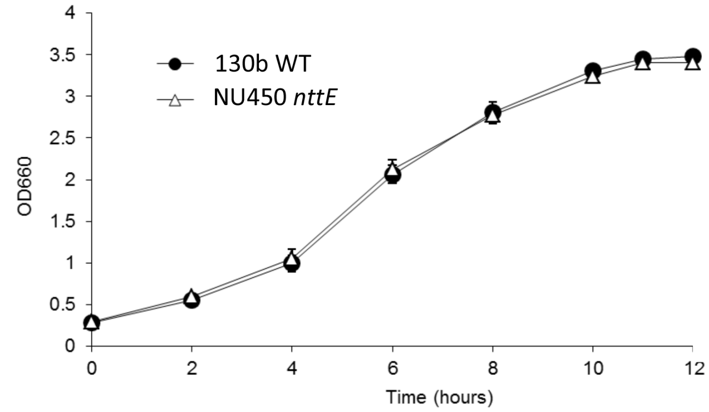

Supplementary Figure 1: Extracellular growth of *L. pneumophila* wild type and *nttE* mutant strains. Wild-type (WT) strain 130b and the *nttE* mutant strain NU450 were inoculated into BYE broth, and then the cultures incubated at 37°C with shaking. At various times post-inoculation, bacterial growth was monitored spectrophotometrically. The data points represent the mean and standard deviation of triplicate cultures, and the results presented are representative of two independent experiments.

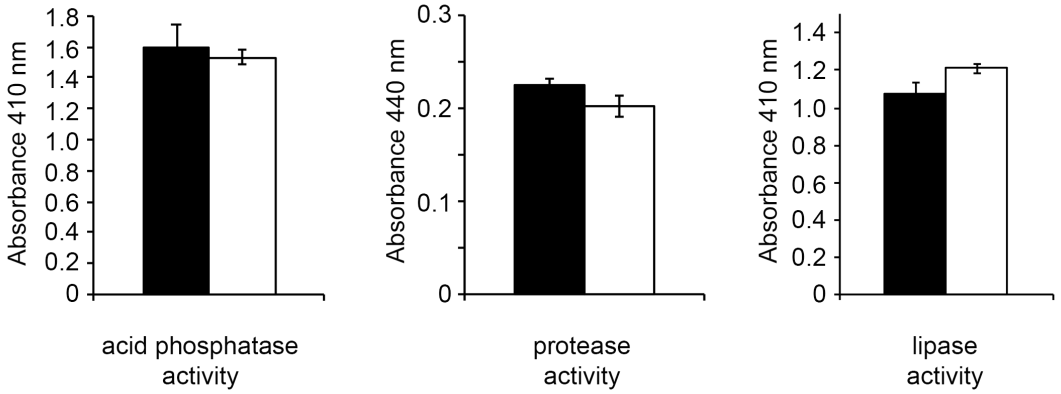

Supplementary Figure 2: Secreted enzymatic activities of *L. pneumophila* wild type and *nttE* mutant strains. Wild type strain 130b (black bars) and *nttE* mutant strain NU450 were grown in BYE broth at 37ºC until late-log phase, and then culture supernatants were filter-sterilized and measured for (A) acid phosphatase activity by *p*NP-phosphate hydrolysis, (B) protease activity by azocasein degradation, and (C) lipase activity using *p*NP-caprylate hydrolysis. Data are the mean and standard deviations of three biological replicates.

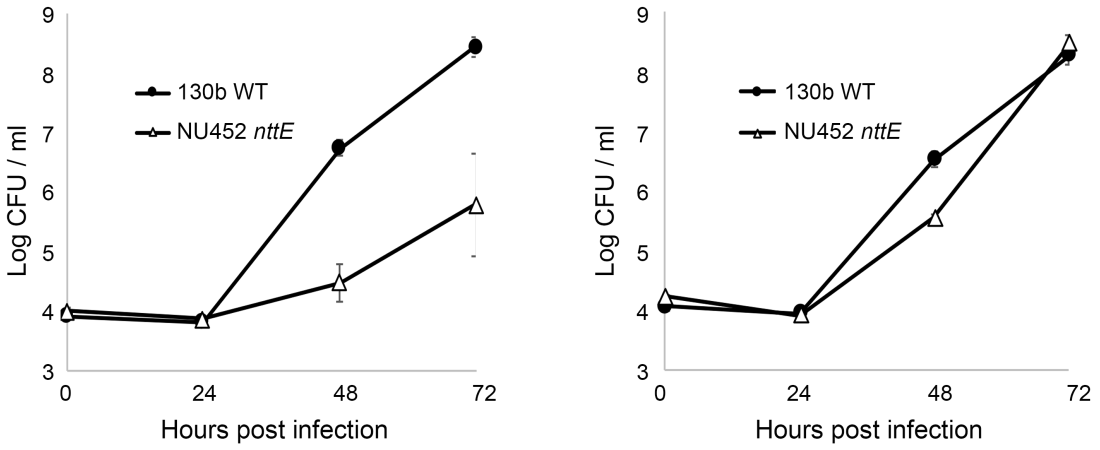

Supplementary Figure 3: Intracellular infection of aquatic amoebae by *L. pneumophila* wild type and *nttE* deletion mutant. *A. castellanii (*left) or *V. vermiformis* (right) amoebae were infected with either the wild-type (WT) strain 130b or the *nttE* deletion mutant, strain NU452, and at the indicated times, CFUs from the infected monolayers were determined. Data are the means and standard deviations from four infected wells. The results in each panel are representative of three independent experiments. The recovery of the mutant was significantly less than that of the WT at 48 h in both types of amoebae ($P < 0.001$) and at 72 h in the *A. castellanii* on the left ($P < 0.01$).

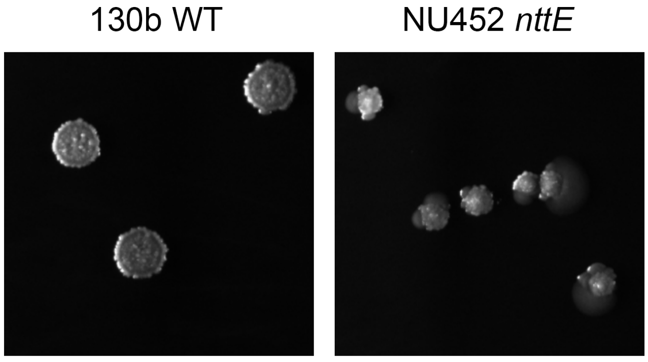

Supplementary Figure 4: Colony morphology of *L. pneumophila* wild-type and *nttE* deletion mutant. Wild-type (WT) 130b and *nttE* deletion mutant NU452 were plated onto standard BCYE agar and then colony morphology was observed after 7 days of growth at 37ºC. Images are representative from platings done on three independent occasions.

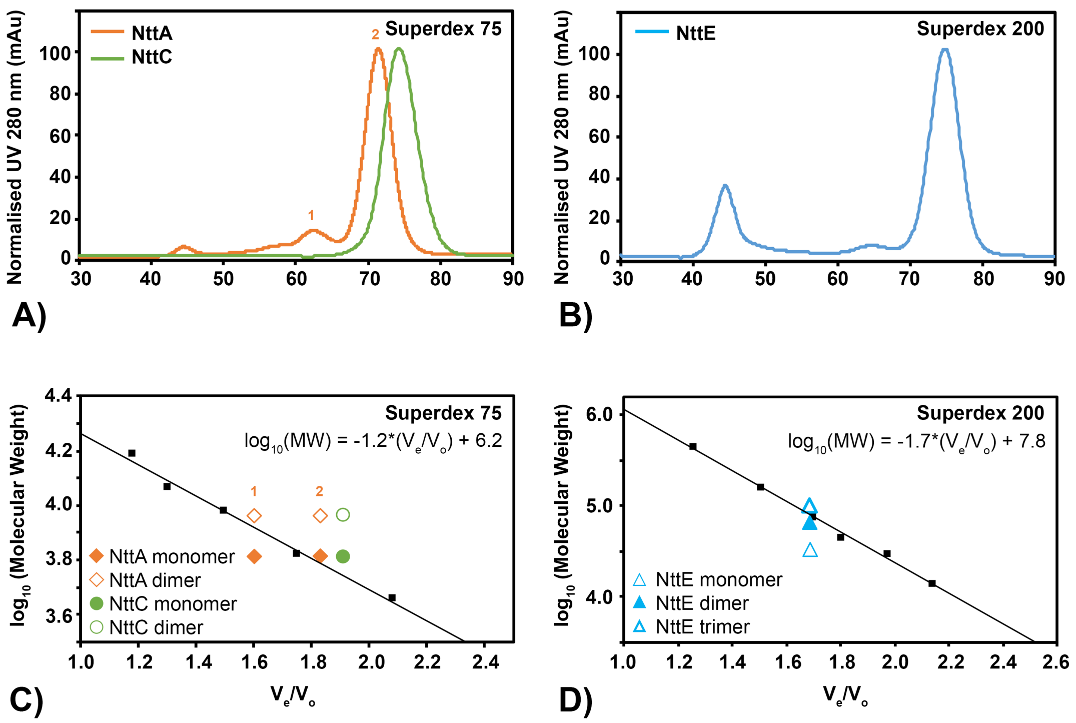

Supplementary Figure 5: Analytical size exclusion chromatography (SEC) of NttA, NttC and NttE. Each trace shows a normalized chromatogram of each substrate injected onto either a Superdex 75 or 200 column, where (A) are the traces for NttA (orange) and NttC (green), and (B) is the trace for NttE. Two peaks in the NttA trace are numbered. (C) The NttA, NttC and (D) NttE V_e/V_o (elution volume/column void volume) were plotted against their Log_{10} molecular weights on a standard curve created using molecular weight standards (GE Healthcare). These profiles show that NttA (peak 2) and NttC are likely monomeric and NttE is likely dimeric or trimeric in solution. A minor dimeric NttA species (peak 1) is also evident.

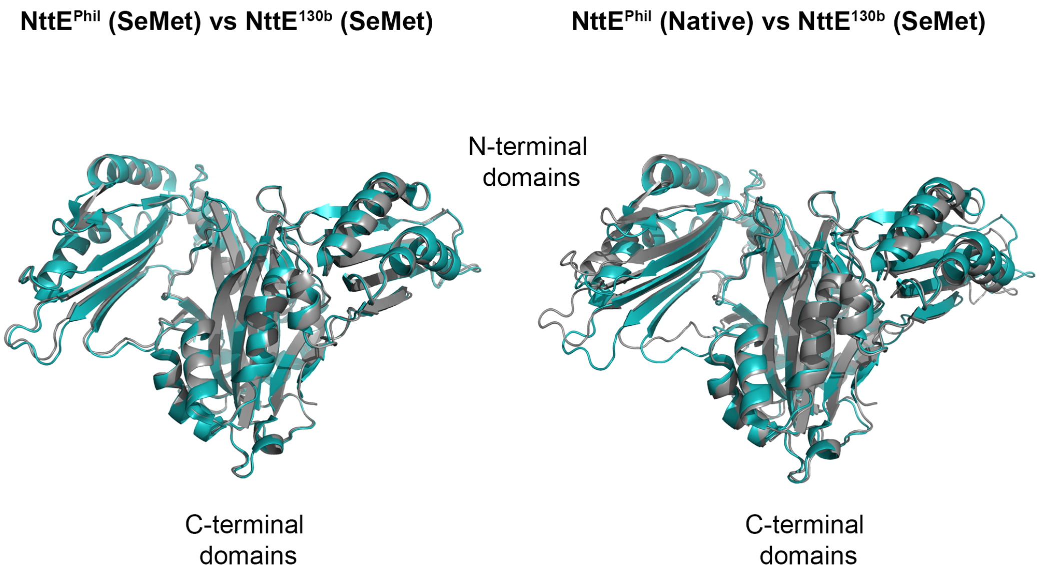

Supplementary Figure 6 Superposition of NttE-Phil crystal structures onto NttE-130b. (Left) Superposition of selenomethionine labelled NttE-Phil (grey) and selenomethionine labelled NttE-130b (teal). (Right) Superposition of native labelled NttE-Phil (grey) and selenomethionine labelled NttE-130b (teal).

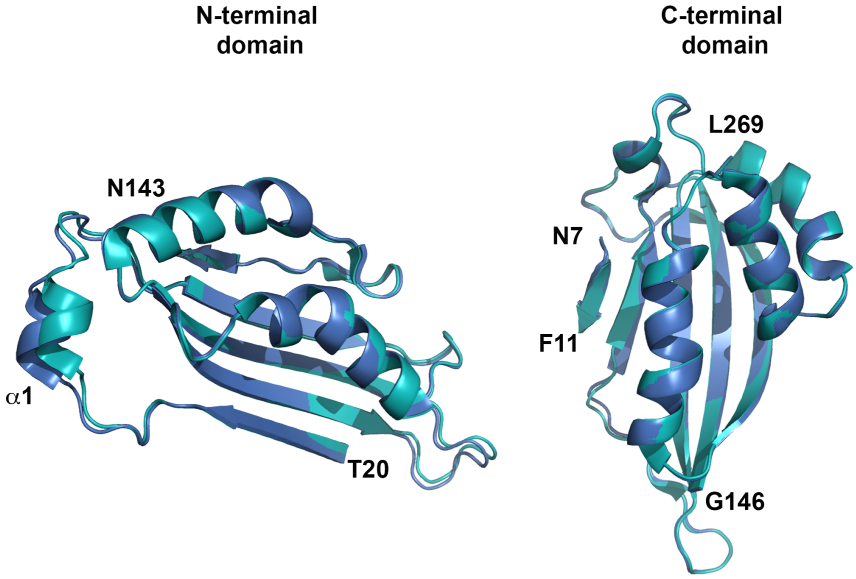

Supplementary Figure 7: Superposition of N- and C-terminal domains from with the NttE-130b dimer. (Left) Superposition of C_α atoms within the N-terminal domain or (right) C-terminal domain. Terminal residues are annotated along with the variable α1 helix in the N-terminal domain. Each chain is coloured blue or teal.

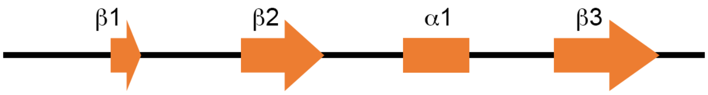


```
L. longbeachae        -------ATEIVVPKLPSPEHTLIKRYSNPINSDSELTIAQRTMDYPTHVVRMEDVQLHD  53
L. sainthelensi       -------TSEIAVPELATPEHTLIKRYSNQVNSDSQLPIAQRTIDYPTHVVRMEEVGLQN  53
L. gratiana           -------STEIVVPKLATPDHTIIKRYSNTADSDSGLPKAQRTLDYPTHVVRMEEVQLQD  53
L. santicrucis        -------ATEIVVPKLATPDHTLIKRYSNEVDSDSGLPIARRTIDYPTHVVRMEKVQLQD  53
L. cincinnatiensis    -------ATEIVVPKLAPPD-TLVKRYSNPINSDSGLPIAQRTIDYPTHVVRMEEIPLQD  52
L. lansingensis       ------------IQNIKTPKHTIIKQQTNVQMIFKNPALMVRTTEYPTQIVRVGG-ELDN  47
L. jordanis           ------ESSHIYSQEISSSKYKIIKKDSNLQTIIKHPEMFTRTHEYPTQIVRIHG-KLNN  53
L. spiritensis        --ESLQTSFDIRMTAPSAGKHTVVKKYSNLKEVMNNHQSLIRNGNYPTQIVRIRG-EFAH  57
L. rubrilucens        ---NLAEPPK-EVITPVFDGQAVVKQYSNFQEAAGHKQWFERHQPYPTQVVRVQG-VLSG  55
L. erythra            ---NIAELPKEEMVAAGLSEHTVVRQYTNFQDVADHKQWFARHQPYPTQVVRVQG-DLGG  56
L. dumoffii           --TANDNKEHLIFSPLPNDKNWIVKHYSNEQEIPNQQHMLQRTVDFPTQIVRVRG-NVAG  57
L. waltersii          --SSDNIKDGFMFSSIPEHKNTVVRHYSNEQKMPDLKQMSQRSIDFPTQIVRVNG-SVAG  57
L. cherrii            --HSDPGLDGLIFSPVTENKNTVVRHYSNEQVRPNLQQMTQRTVDFPTQIVRVRG-NVKG  57
L. steigerwaltii      --NSDQRLEGLIFSPLAKDKNTVVRHYSNEQEVPNLNQMMQRTIDFPTQIVRVRG-SVAG  57
L. pneumophila        --NSDDNADGLIFSPLPQNKNTVVRHYSNEQEMPNLSQMAQRTIDFPTQIVRVSG-NLTG  57
L. steelei            --NSDHNLEGLIFSPLPKNKNTIVKHYSNEQEIPDLQQMAQRTVDFPTQIVRVRG-NVEG  57
L. anisa              ----DNSTEGFIFSPLPKNKNTVVRHYSNEQKLPDLKQMSQRTVDFPTQIVRVRG-NVAG  55
L. parisiensis        --HADSNSEGLIFSPLPKNKNTISRHYSNEQELPDVQQMIQRSIDFPTQVVRVRG-NVAG  57
L. bozemanae          --HTDSNSEGLIFSPLPKNKNTVSRHYSNEQELPDLQQMVQRTIDFPTQIVRVSG-NVAG  57
L. tucsonensis        --HSESGSEGLIFSPLPKNKNTVSRHYSNEHELPDLKQMVQRTIDFPTQIVRVRG-NIEG  57
L. quinlivanii        --DEVPENKPVIPAANAAPENTVLRQYTTEDKTADL-ASFQRSIDYPTQIVRIKG-SLAD  56
L. birminghamensis    --DTDLNSQSIIAVTPSVDKNTVVRQYSTEDKATDYQKNLQRSIDYPTQIIRISG-SVSD  57
L. shakespearei       HT-EPSTDKLRFLQNPV-SKNTIVNTYSNESEGLNQLQSLTRTVDFPTQIIRISN-ELKG  57
L. worsleiensis       QV-MLHTDR-MTPGNNLSPRNSIEKTYANIPNALHEQQAFTRTMDYPTQIIRMSA-SMEG  57
L. quateirensis       QS-ELPTDSMRLTHNPL-SKNSIQKIYSNTTENLNQLQSFTRSADYPTQVIRMSG-TMEN  57
L. moravica           HS-EHPSDTLILDHNPLLSKNSIQKTYSNSTERLNQLQSFTRSMDYPTQIIRMSV-SMEN  58
L. gormanii           QTLDDDVTTLT-ISQNPENKNSQVITYSNETDNLRQNKPFTRSIDYPTQIIRMEK-NIES  58
L. drancourtii        -YPKDEFNSMT-LAPKIENQNTHTATYSNESKGYAQIKSFNRSIEYPTQIIRMES-NIEN  57
A. siphonis           --AAIDQPIKITAMARPLSPNTIIREHSSLKEGAARATLPKRTTDYPTQIVRMEA-ELKG  57
```

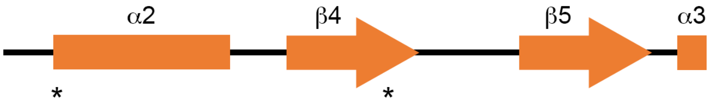


```
                          *                            *
L. longbeachae        SALNCEQVHQKIDEFFVKKLPVNMIYYNIITYCSYDAENP-NIAKNYTINAYFDPVTDQA  112
L. sainthelensi       TTLNCEQVHQKIDEFFVKKLPVTMIYYNIIAYCSYDVENP-DVAKNYTINAYFDPVTDPA  112
L. gratiana           TSLNCEQVHQKINEFFVKKLPVAMTYYNIIAYCSYDGENP-NIAKNYTINAYFDPLTDQA  112
L. santicrucis        STLNCEQVQQKINEFFVEKLPVTMIYYNIITYCSYDAENP-DIAKNYTINAYFDPVTDQA  112
L. cincinnatiensis    PTLNCEQVHQKINEFFVKKLPVTMIYYNIIAYCSYDAENP-DIAKNYTINAYFDPVTDQA  111
L. lansingensis       STLTCDEVEKEIDSFFSNQIKHDLFYYNTLIMCGYDPK--TNYAIRYSIQSYFDPLNDKG  105
L. jordanis           STLNCDEVAKTIDDFFSKAIKHDLFYYNTLIICGYDPK--TDYAISYNLQSYFDPLNDAA  111
L. spiritensis        PSRYCDEVLGEIDEFFNRKITSDQFLYNTLVFCGFDPA--TEYATQFAINSYFDPLNDKA  115
L. rubrilucens        -ERHCEAILQEIDDFFVSKITHEKFLYNTLVFCGYDPE--TEYAVQFAINSYFDPLNDGA  112
L. erythra            -ERHCEAVLKEIDDFFVSRITHEKFFYNTLVFCGYDPK--TEYAVHFAINSYFDPLNDGA  113
L. dumoffii           LELSCEEIGDEIEQIFTNKISPDLFLYNTYINCAYDQDSPEEYAVSFSIQSYFDPLTDKA  117
L. waltersii          LELSCDEVENEIDHVFSEKILPDLFTYNIYVNCAYDYKSPEQYAVSFSIQSYFDPLTDEG  117
L. cherrii            MGLSCEEVENEIERVFSKKITPDLFTYNTYINCSYDQESPDEEAVSFSIQSYFDPLTDKA  117
L. steigerwaltii      LGLSCDEVEDEIDRVFSKKISPNLFTYNTYINCGYDQDSPDEDAVNFSIQSYFDPLTDKA  117
L. pneumophila        LELSCDDVENEIDQVFSKKISPNLFTYNTYVSCGYDVNDPEQHATNFSIQSYFDPLTDNA  117
L. steelei            LGLSCEEVTDKIDQVFSEKISPNLFTYNTYVNCGYDQDSPEEYATSFSIQSYFDPLTDKA  117
L. anisa              LKLSCEEVEDEINKVFSKKISPNLFTYNTYVSCGYDQDSPEQYAVSFSIQSYFDPLTDKA  115
L. parisiensis        LKLSCEEVEDEIDRVFSKKITPNLFTYNIYVNCGYDQDSPEQHAVNFSIQSYFDPLTDKA  117
L. bozemanae          LKLSCEEVEDEIDRVFSKKISPNLFTYNTYVNCGYDQDSPEQFAVNFSIQSYFDPLTDKA  117
L. tucsonensis        FKLSCEEVEDEIDRIFSKKISPNLFTYNTYVNCGYDQDSPEQYAVNFSIQSYFDPLTDKA  117
L. quinlivanii        KNLSCDEVNKAIDKAMVSKITPDKFTYNIYISCSYDPQ--TRLATSFLINTYFDPLSDNA  114
L. birminghamensis    QNISCDQVNGAIDKAFVSRITADKFTYNIYISCSYDPQ--THLATSFLINAYFDPLSDNA  115
L. shakespearei       KEITCEQVNAQIEETVFSHITSDKFIYNILISCGYDPD--TNYATSFFINSYFDPKTDEA  115
L. worsleiensis       QQLTCDQVNEAIEEKIIRFITTDKFSYQTYFSCTYNPD--THYAIHFKINSYFDAINDEA  115
L. quateirensis       QELTCDQVNEAIEEKIVDYITSDKFSYQTYFSCTYNPD--TNYAISFQINSYFDPINDEA  115
L. moravica           QQLTCDQVNEAIEERIVHYITSDKFSYQTYFSCTYNPN--TNFAINFQINSYFDPVNDEA  116
L. gormanii           QQLSCDEVNNQIDKILVQHIANEQFTYAIYISCYYNPQ--TKLATQFIISSYFDPVSDQA  116
L. drancourtii        QQITCAAVHEQINKTLVENIANEQFTYAIYISCHYDPE--TFLATQFTINSYFDPVNDEA  115
A. siphonis           PPQNCDEVFQKIDEFFNDHITYDRFFYNTINYCVYDPN--TNFAKKFIINSYFDPLDDEA  115
```

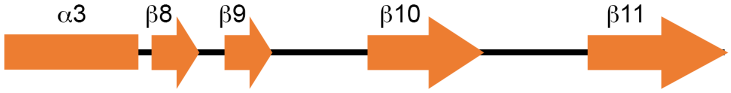

L. longbeachae	IEYLKNYIHEYNGQDLMG-VPFNIEEVKKVIVSLNFDAGIRKDKFGQIILRYFHENQTHS	171
L. sainthelensi	IEYLKNYIHEYNGQDLMG-VPFNIEEVKKVIVSLNFDAGIRKDEFGQIILRYYHENQTHS	171
L. gratiana	IEYLKNYIHEYNGQDLMG-TPFNIEEAKKVIVSLNFDAGIRKDEYGQIILRYYHENQTHS	171
L. santicrucis	VEYLKSYIHEYNGQDLMG-APFNIEEAKKVIVSLNFDAGIRKDEYGQIILRYYHENQTHS	171
L. cincinnatiensis	IEYLKNYIHEHNGQDLMG-APFNIEEAKKVIVSLNFDAGIRKDEYGQIILRYYHENQTHS	170
L. lansingensis	IEYLQSYLAEHNGKMLLG-WPFYVEDAQGVVVSMSMNAGRKNGHNDSTMLVLRHDNANHY	164
L. jordanis	IDYLQTYLKEHNGKLLLG-KTFFVENAEGVVISLNIHVGNKNAPSEQSMLLLRTDNSNHY	170
L. spiritensis	VAYLQTYLAEHNGRELLG-TTFQVEEAKGVAVSMDIDAGEEADRSSQVLTRYRHDNQTHY	174
L. rubrilucens	IYYLENYLRSHNGQDLLG-VPFEVESARGVAVSLNIDAGVLNHRNDSTMLRYRHDNQSHY	171
L. erythra	IYYLENYLGSHNGQDLLG-VPFEVESAIGVAVSLNIDAGVLNHRNDSTLIRYLHDNQSHF	172
L. dumoffii	VDYLKSYLQEYNGYNLFDTSTLHIENAKGIIVSMTLNAGLKHNPEQASFTLYRQDRKNFY	177
L. waltersii	IEYLKTYLKEYNGYNLFDTTPLQIENAKGIISSINLNAGLKSNPDKTPLMLYSQDRSNIY	177
L. cherrii	VDYLKAYLQEYNGYNLFNASTLDFESAKGVIVSMNLNAGLKNNPNQVPMMLYRQDRNNLY	177
L. steigerwaltii	VDYLKSYLQEYNGYNLFNTSTLQIENAKGVIISMNLNAGLKSNPDKIPLMLYRQDRNNIY	177
L. pneumophila	VDYLKSYLKEYNGYNLFNTTTLQIENAKGIIVSMNLNAGLKSNPDKTPFTLYRQDRNNFY	177
L. steelei	VDYLKSYLKEYNGYNLFNTATLHIENAKGVIISMNLNAGLKNNPDKVPLMLYRQDRSNFH	177
L. anisa	IDYLKAYLQEYNGYNLFNATTLQIENAKGVIISMNLNAGLKSNPDKTPFTLYRQDRNNFY	175
L. parisiensis	IDYLKAYLQKYNGYNLFNVATLHIENAKGVITSMNLNAGLKNNPDKPPFALYSQDRSNFY	177
L. bozemanae	VDYLKAYLQEYNGYNLFNAATLHIENAKGVIISMNLNAGLKNNPDKPPFTLYRQDRNNFY	177
L. tucsonensis	VDYLKAYLQEYNGYNLFNATTLHIENARGLIISMNLNAGLKNNPDRPPLTLYRQDRNNFY	177
L. quinlivanii	IDYLNNYLAEYNGSDLLG-TSFNIESAKGVVISMNLNAGYKNNMRKTTYTLYHQDRGNYF	173
L. birminghamensis	IDYLNNYLAEYNGSDLLG-TRLDIESAKGVIISMNVNAGYKNSTRKTTYTLYHQDRGNYF	174
L. shakespearei	IDYLKDYLSQNNGTDFLG-TKLNIESAKGVVIALNIAAGMKKKPNTPPFIEYQQDRSNFY	174
L. worsleiensis	VTYLQSYLNANNGSEFLG-THLNIEPAQGLVIALTFSAGTKKNPTNPPFIEYRQDRANFY	174
L. quateirensis	VSYLKSYLSANNGSEFLG-TQLNIESAKGLVVALNFSAGMKKNPKNPPFIEYRQDRSNFY	174
L. moravica	VNYLKSYLSANNGSDFLG-TQLNIESAKGLVVALNVSAGMKKNPKNPPFIEYRQDRSNFY	175
L. gormanii	ITYLESYLNEYNGTNLLG-TEYKIESAKGLIISLDIAAGIKKKPNRPPFTEYRKDHSSFY	175
L. drancourtii	ITFLKNYLSEYNGSTLLG-TKFKIESAKGLIISLSMAAGMRKNPTKPPFIEYRRDRSNFY	174
A. siphonis	ITYLETYLAQHNGRDLLG-STFHVENAQGLIVSLNIDSGLEDSRNASTLLRLQHDNSSHY	174

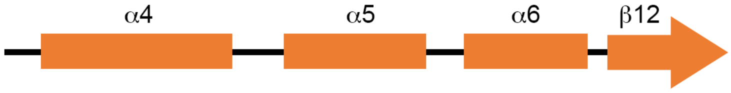

L. longbeachae	FQNFFDVRRGLIADIHRRINSNERDTIIPLFTKWFSPGGESLYIQTLKRSDYLLLQPELI	231
L. sainthelensi	FRNFFDVRKELITDIHHRINSNEKDTIIPLFTKWFSLGGESLYTHVLKKSDYLLLQPELI	231
L. gratiana	FQNFFDVRKELITDIHHRLHSNESATIIPLFTKWFSPGGELLYTHILRKSDYLLLQPELI	231
L. santicrucis	FQNFFDVRKELIADIHRRINSNESATIIPLFTKWFSPGGESVYTHILKKSDYLLLQPELI	231
L. cincinnatiensis	FQNFFDVRKELITDIHRRINSNESATIIPLFTKWFSPGSESLYTHILRKSDYLLLQPELI	230
L. lansingensis	FANNYQLLKELVADIHQRYNSNNPELILPFLDRWFFSFAGMVYERILKNSTYTELQPERI	224
L. jordanis	FANNYQVMKELMADIKERYNSNDPELILPFLSKWFYPSAGMFYQRLLKLSNYTELLPERI	230
L. spiritensis	FSSNYDLTKQLITDIRQRFYSNDPDKILPFLQKWFFHNAGSMYYYVLKSSNFVELRPQRL	234
L. rubrilucens	FASNYDLMNDLIHDIYERFYSNDPDLILPFLNHWFYAFADRTYSAVLSHANYVELQPERL	231
L. erythra	FASNYDLMKELIQDIYERFYSNDPDLILPFLNRWFYSYADRTYDSILSRVNYVELQPERL	232
L. dumoffii	FKSNFDMRKELIADIYHRFYSNNPEVILPFFEKWISPAANTIYHAILKASNYLELQPERI	237
L. waltersii	FKSNFEMRKELINDIYQRFYTNDPEVILPFLDKWIYPSAGTVYYSILQASNYLELQPERI	237
L. cherrii	FKSNFEMRKELIADIYQRFYSNNPEVILPFLDKWIFSFAGTIYHSILKASNYLELQPERI	237
L. steigerwaltii	FKSNFEMRKELIVDIYQRFYSNDPEVILPFLDKWIFSSAGAIYHSILKASNYLELQPERI	237
L. pneumophila	FKSNFDVRKELISDIYQRFYSNDPDMILPFFDKWIFSYAGSVYYSILMASNYLELQPERI	237
L. steelei	FKSNFDMRKELIADIYQRFYSNAPEIILPFLNKWLFSYAGTVYYPILQASNYLELQPERI	237
L. anisa	FKSNFDMRKELIADIYQRFYSNDPEIILPFLDKWIFSYAGTVYYSILKASNYLELQPERI	235
L. parisiensis	FKSNFDMRKELIADIYQRFYSNDPEMILPFLDKWIFSYAGAVYYSILKASNYLELQPERI	237
L. bozemanae	FKSNFDMRKELITDIYQRFYSNDPEMILPFLDKWIFSYAATVYYPILKASNYLELQPERI	237
L. tucsonensis	FKSNFDMRKELIADIYQRFYSNDPEMILPFLDKWISSYARAVYYSILKASNYLELQPERI	237
L. quinlivanii	FKSNYDLKKELIADIYSRFYSNDPEVVLPFLDKWISPYASSIYPGILRSSNYVELQPERI	233
L. birminghamensis	FKSNYALKNDLLADIYNRFYSNDPSVVLPFLDKWINPYASNIYPGILRSSNYVELQPERI	234
L. shakespearei	FSSNYEMSNKLFTDIYQNFFSNDPEKILPFLDRWVFSFAGSVYQAALKDSNYVELQPERI	234
L. worsleiensis	FKNNYEMNKHFLTAIKNNFYSNEPEKILPFLDRWLFPHAGTVYKAVLRDANYTELRPEQI	234
L. quateirensis	FKNNYEMNKKLFTDINQNFYSNDPEKILPFLERWLFPYAGTIYKAVLRDSNYAELQPEQI	234
L. moravica	FKNNYEMTKKLFTDINQNFYSNDPEKILPFLDRWLFPHAGPIYKAVLRDSNYAELQPELI	235
L. gormanii	FKSNYEMKSKLFSDIYQNFFTTDPDKILPFLDKWVSSHASSFYKAVLRDSNYVELQPEKI	235
L. drancourtii	FKSNYEMRSKLFADIYQNFYSNNPAQVLPFLNKWVFSHADSMYKAILRDSNYAELQPERI	234
A. siphonis	FTSNYAMQSDLISDVRQRFYSNDPGLILPFIDKWFFT-SGWIYERVLKNSDYVELQPELI	233

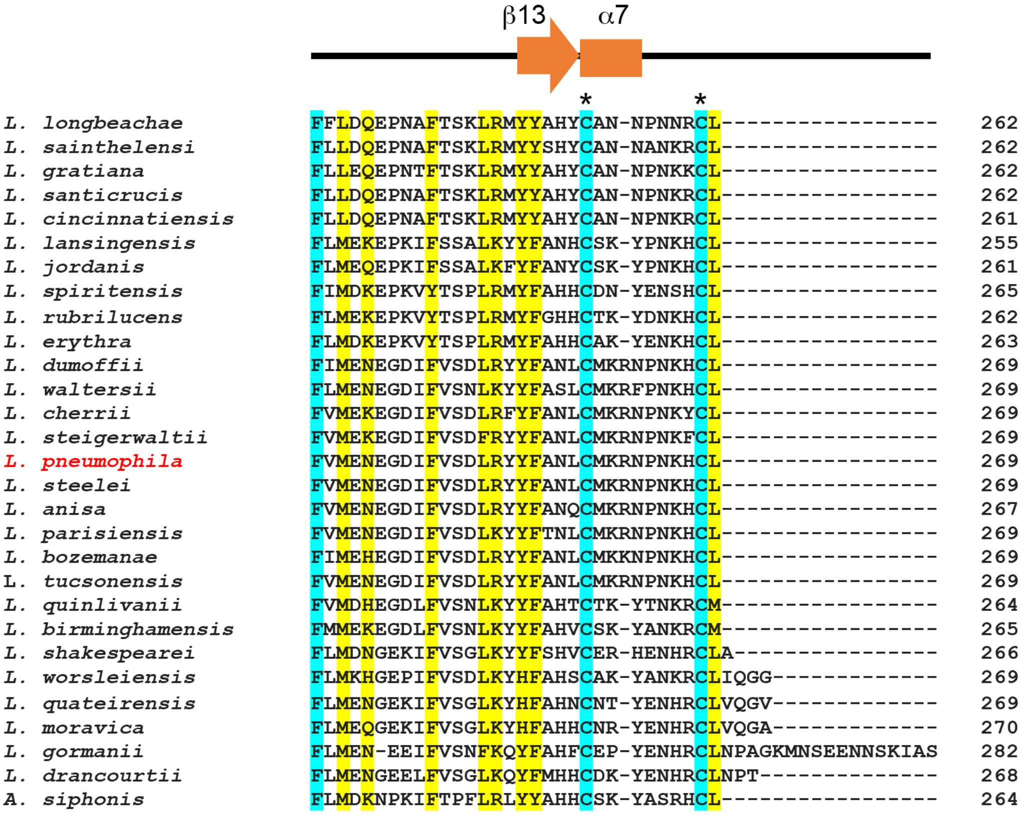

Supplementary Figure 8: Sequence alignment of *L. pneumophila* NttE and other NttE-like proteins. Secondary structure elements of NttE are shown above a (rectangle: α-helix; arrow: β-strand). Amino acid identities and similar residues are indicated by background shading in cyan and yellow, respectively. Disulfide forming cysteine positions are indicated by an asterisk. *L. pneumophila* is coloured red.

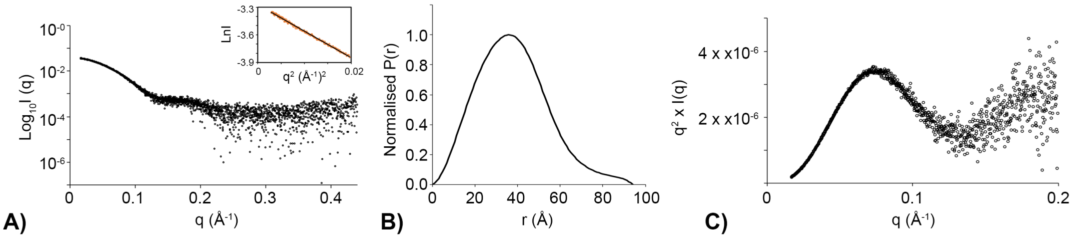

Supplementary Figure 9: SAXS analysis of NttE-130b. (A) Experimental scattering curve of $NttE^{130b}$ (black open circles). Inset: Guinier Region (orange open circles) and linear regression (black line) for R_g evaluation. (B) Shape distribution [P(r)] function derived from SAXS analysis for NttE-130b. (C) Kratky plot indicates that NttE-130b has dynamic features in solution.

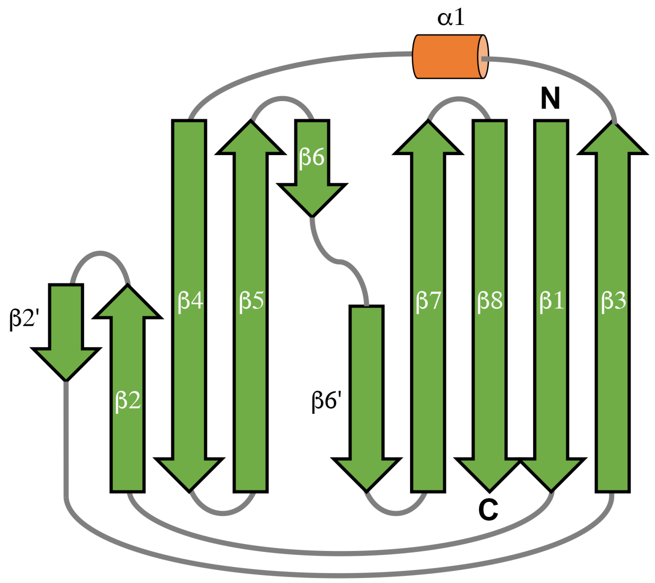

Supplementary Figure 10: Topology of NttC. Schematic representation of NttC with secondary structure strands in green, helices in orange and loops in grey. β-sheets are labelled 1–8 and helices as α1.

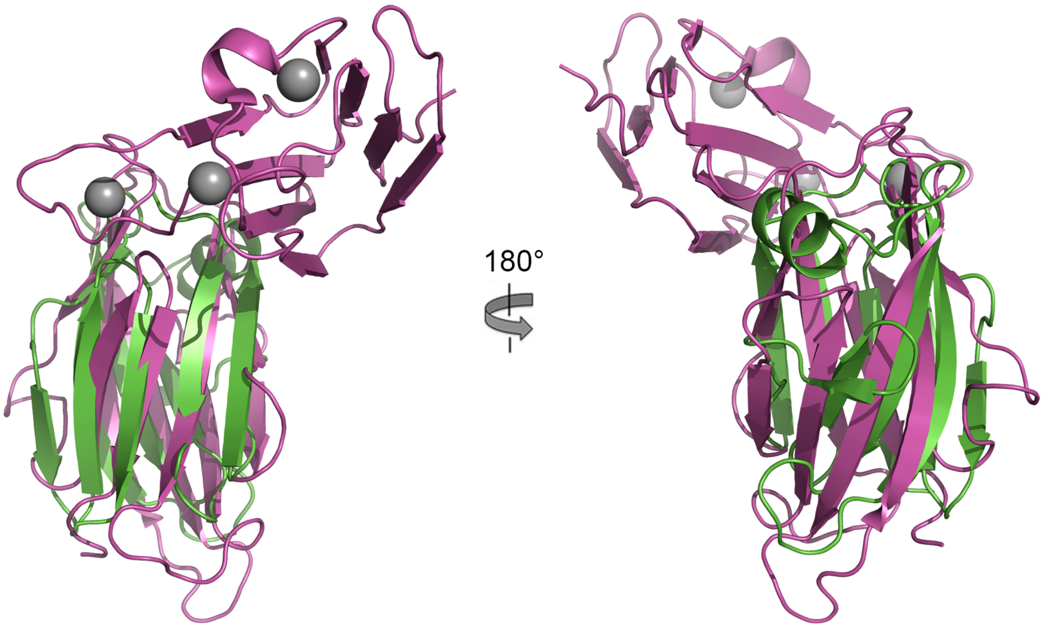

Supplementary Figure 11: Superposition of *L. pneumophila* 130b NttC on *D. discoideum* DdCAD-1. NttC is coloured green and DdCAD-1 (PDB ID code 1B1O) is magenta. Ca^{2+} bound to DdCAD-1 are shown as grey spheres.

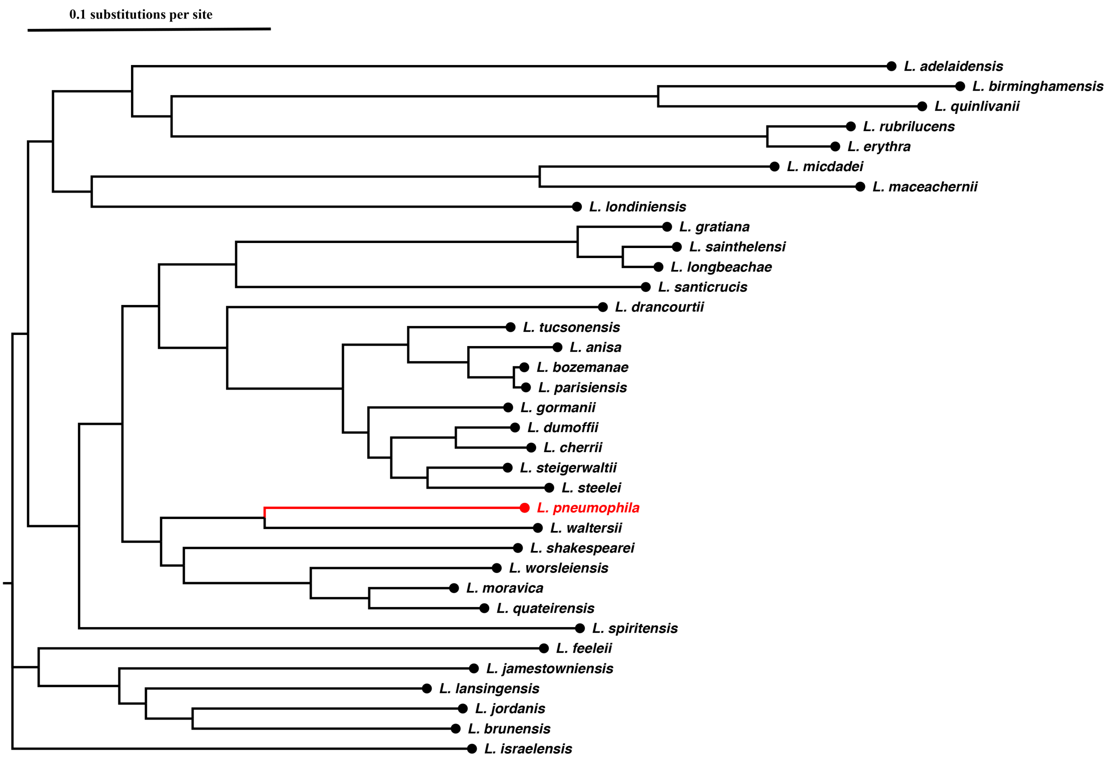

Supplementary Figure 12: Phylogenetic tree of NttC-like proteins. Scale bar indicates the number of amino acid substitutions per site. *L. pneumophila* is coloured red.

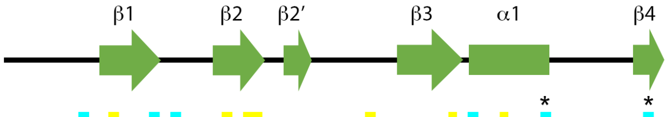


```
L. adelaidensis        TTLPTVPPHNLTTHNLTNVWSEAFVEGTIRTEKNATPPNTTKKVAWTEVLFICHKR----PQCW  60
L. birminghamensis     ------SPEHMIIHNKTDLISNAFVDEM-PG-PRPTLAHSTSRVLWFIVQTGCRKHKA-EGICP  55
L. quinlivanii         ------SPESMIIHNTTNLISNAFVDEM-PG-PRPTLPHSTSKVSWFIVQGGCRKHKT-E-ACP  55
L. micdadei            ------NPTNLVIRNTTKVEMNEYIDGIIP--AIPTPPESKGTVPWNLIRMACYGHTV-NNKCS  55
L. maceachernii        ------HPTDLIIRNTTDVEMNEYIDGIVP--AIPTPAKSVGTVPWTLIEMACYGYTL-KGKCS  55
L. rubrilucens         ------APSHLTTDNQTNYQSNAFINGTVQS-PYPTPKKTKKSLLWSLVQLACAPYVDSQKQCG  57
L. erythra             ------APSHLTTDNQTNYQSNAFINGTVQS-PYPTPKKTKKKLLWSLVQLACAPYVDSQKQCG  57
L. feeleii             ------SPTYLIIHNRTNVESNAYIDGTIDS-HQPTKANSDGKVHWAVVRMICFGHIT-GGNCG  56
L. jamestowniensis     ------SPAYLVTHNKTNVESNAYVAGKA-S-PHPTAPKSDGKVSWYVVRMACFGHTD-KNICK  55
L. lansingensis        ------SPAYLITHNRTNVESNAYVAGVP-S-PHPTKANSDGKVSWLAVRVACYNHTE-NGICK  55
L. jordanis            ------SPAYLITHNKTNVESNAYVAGVP-S-PHPTRANADGKVSWLAVRLACNGHVNKEGRCA  56
L. brunensis           ------SPAYLVTHNKTNVESNAYVAGVA-A-PHPTAPNSDGKVSWLVVRMACYGHVDKSGRCH  56
L. gratiana            ------APEHLITHNNTSEESNAYIAGA-PS-LYPTPANTTKKVFWNLVKLACYGHTT-NGKCS  55
L. sainthelensi        ------APEYLTTHNNTSEESNAYIAGA-PS-LYPTPANTTKKIFWNLVKMACYGHTT-NGKCS  55
L. longbeachae         ------APEYLTTHNNTSEESNAYIAGA-PS-LYPTPANTTKKVFWNLVKMACYGHTT-NGKCS  55
L. santicrucis         ------APEYLITHNYTSEESNAYIGGVVAS-PYPTAANITQKVYWNLVKLACYGYTSPDKKCT  57
L. drancourtii         ------SPAYLTTYNNTSEESNAYVAGV-PS-IYPTPANSKRDVYWNLVKLACYGHTT-GRNCL  55
L. tucsonensis         ------SPVYLITHNNTPVESNAYIAGV-PS-PYPTAANSTQKVYWNLVKLACYGHTT-GKQCT  55
L. anisa               ------SPVYLITHNNTPVESNAYIAGV-PS-AYPTPANSTQKVYWNLVKLACYGHTT-GKQCS  55
L. bozemanae           ------SPVYLITHNNTSVESNAYIAGV-PS-AYPTAANSTQKVYWNLVKLACYGHTT-GKQCS  55
L. parisiensis         ------SPVYLITHNNTSVESNAYIAGV-PS-AYPTAANSTQKVYWNLVKLACYGHTT-GKQCS  55
L. gormanii            ------SPTYLITHNNTPEESNAYIAGV-PS-AYPTPANSTQKVYWNLVKLACYGHTT-GKQCS  55
L. dumoffii            ------SPAYLITHNKTPEESNAYIAGV-PS-AYPTAGNSTQKVYWNLVKLACYGHTT-GKQCT  55
L. cherrii             ------SPAYLVTHNNTPEESNAYIAGV-PS-AYPTAGNSTQKVYWNLVKLACYGHTT-GKQCT  55
L. steigerwaltii       ------SPAYLITHNKTPEESNAYIAGV-PS-AYPTAANSTQKVYWNLVKLACYGHTT-GKQCA  55
L. steelei             ------SPAYLITHNKTPEESNAYIAGV-PS-AYPTAGNSTQKVYWNLVKLACYGHTT-GKECS  55
L. londiniensis        ------APQKLITHNTTDYESNAYVAGIVPS-THPTKAHSKGVVSWVEVRMACFGHDA-GGKCS  56
L. israelensis         ------SPNYLITHNKTNLESNAYVANSIPS-RHPTPAHSDGKVSWTLVKIACFGRST-NGVCP  56
L. spiritensis         ------GPSYLVTHNDTSVESKAYVAGIIPA-PTPAAPNKTTSISWIVVKMACYGHTT-NNKCP  56
L. pneumophila         ------APAYLTTHNRTGEESNAYIAGSIPS-LYPTAAYSTNQVYWNLVRLACYGHTT-NGQCP  56
L. waltersii           ------APLYLTTHNNTNEESNAYIAGTIPS-PYPTAAHSTGQVYWNLVKFACYGHTT-NGKCS  56
L. shakespearei        ------VPAFLTTHNNTDVESNAYVAGTIPS-PHPTKAHSTRQVPWNMVRIACYGHTS-AGKCS  56
L. worsleiensis        ------VPTYLITHNNTNVESNAFIAGTIPS-PYPTPANSTRQVYWNMVKMACYGHTT-NGRCS  56
L. moravica            ------VPTYLTTHNNTNVESNAYVAGTIPS-PYPTPANSTRQIYWNMVKMACFGHTT-NGKCS  56
L. quateirensis        ------VPVYLTTHNKTNVESNAYVAGTIPS-PYPTPANSTRQIYWNMVKMACYGHSS-NGRCS  56
```

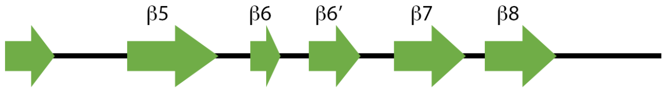


```
L. adelaidensis        ALVKMNTKGDKSVDLCKVTLDVKTGIITPEALCAGNNGYALTVNGPGEVTLTRK---------  114
L. birminghamensis     AVIKMGIDRDKPILVGTLYIDISSGEITPARIS-A-NGFNVDVDAPGEITISEDK--------  108
L. quinlivanii         AVFKMAIDKAKPIVVGTLYIDIITGVITPVHLE-G-NGFTIDVDAPGEITISAD---------  106
L. micdadei            ALIKLATDTNRPFNLGTITIDIETGEISPKSIT-GPKGYKFTVTGAGEATLTVSAQSVQRKAH  117
L. maceachernii        ALIKLATDTNKPFKLGTITIDMETGEISPQSIT-GPKGYKFKATGPGKATLIQE---------  108
L. rubrilucens         ALIKMGTDTSNPVEVGWLKMNMSTGDITPKKLS-A-NGFTITVVNAGEVIITEDKN-------  111
L. erythra             ALIKMATDTSNPIEVGWLKMNMSTGEITPKELS-A-NGFKITVINAGEVIITEDKN-------  111
L. feeleii             ALIKMATNTPNPVNLGWLNLNIESGVITPSEIR-A-NGYTIKVNGPGEATITKD---------  108
L. jamestowniensis     AVIKMATDTPNPVDIGEVSLNVETGEISPTSLS-R-NGYTITVNGPGEATLTKN---------  107
L. lansingensis        AVIKMATNTPNPIELGVVSMNIESGDITPKALT-G-NGYTMTVNGLGEATLTKN---------  107
L. jordanis            AVIKMATDTASPVDLGTVSVHVDTGDIIPKQIS-G-NGYTLTVNGPGEATLTKN---------  108
L. brunensis           AVIKMATNTDKPIELGTVSMDVENGDIQPKQVT-G-NGYTLTVNGPGEATLTKN---------  108
L. gratiana            ASIKMATNTPNPIDIADVTLDLNSGTITIDKQK-D-YNYIFTINGPGEATIEKR---------  107
L. sainthelensi        ASIKMATNTPNPIDIADVTLDLNSGTITVDKQK-N-YNYIFTINGPGEATIDKR---------  107
L. longbeachae         ASIKMATNTPDPIDIADVTLDLNSGTITVVKQK-N-YNYTFTINGPGEATIDKR---------  107
L. santicrucis         ALIKMATNTPNPINVGTLTLDLNSGDITPKELI-N-NGYHMVINGLGEASITKLP--------  110
L. drancourtii         AVVKMATNTANPIEIGTLSMNLDTGDITPKQLS-N-NGYTVTVTGIAETTITKN---------  107
L. tucsonensis         AIIKMATNTANPIEVGELRMDLETGDITPKYLS-R-NGYTLFVNGLGEATITKN---------  107
L. anisa               AIIKMATNTANPVEVGEVHMDLDTGDINPKYFS-K-NGYTFFVNGLGEATIVKN---------  107
L. bozemanae           AIIKMATNTANPIEVGEVHMDLDTGDINPKYLS-K-NGYTLFVNGLGEATIIKN---------  107
L. parisiensis         AIIKMATNTANPIEVGEVYMDLDTGDINPKYLS-K-NGYTLFVNGLGEATIIKN---------  107
L. gormanii            AVIKMATNTSNPIEVGEVRMDLDTGDITPKFLS-K-NGYNLTINGPGEATITKK---------  107
L. dumoffii            AIIKMATNTSNPVEVGEVHMDLDSGDITPKHLS-R-NGYTLTVNGPGEATITKG---------  107
L. cherrii             AVIKMATNTSNPVEVGEVHMDLDSGDITPKYLS-K-NGYTLTVNGPGEATINKG---------  107
L. steigerwaltii       AVIKMATNTANPIEVGEVHMDLDTGDITPKSLS-K-NGYTLTVNGAGEATIEKR---------  107
L. steelei             AIIKMATNTSNPIEVGEVKMNLDTGDISPKFLS-K-NGYTLTVNGAGEATIEKR---------  107
L. londiniensis        ALIKMETNTSHPVDIGWVTLDLNTGEITPKVVT-G-NGYRLTVNGIGETTLSKD---------  108
L. israelensis         AMIKMATNTPQPFDLGMVYLNLSTGDITPKVLS-A-HGYKMTVNGPGEATLTQN---------  108
L. spiritensis         ALIKMATNTAHPVELGTVEMDLDSGDITPKKLS-A-NGYTLTVNGPAEATLTQN---------  108
L. pneumophila         ALIKMATNTANPIDIGYVTMDLNTGDITPKTLS-A-KGYSLRVIGPGEAEITKN---------  108
L. waltersii           ALIKMATNTANPIDIGYVSLNLDTGDIEPKVLS-A-QGYTLKAIAPGEAEISKD---------  108
L. shakespearei        ALIKMATNTATPVDIGYVSMHLDSGTITPQKIS-G-NGYTLTVNGPGEATITKDAE-------  110
L. worsleiensis        ALIKMATNTSAPIELGYVSMELDSGDISPKVLS-A-KGFTLTVNGPGEATITRN---------  108
L. moravica            ALIKMATNTSSPIDIGYVTMELETGDITPKVLS-A-KGYTLTVNGPGEATITKD---------  108
L. quateirensis        ALIKMATNTSSPIDIGYVSMELDTGDITPKVLS-A-KGFTLTVNGPGEATITKD---------  108
```

Supplementary Figure 13: Sequence alignment of *L. pneumophila* NttC and other NttC-like proteins. Secondary structure elements of NttC are shown above a (rectangle: α-helix; arrow: β-strand). Amino acid identities and similar residues are indicated by background shading in cyan and yellow, respectively. Disulfide forming cysteine positions are indicated by an asterisk. *L. pneumophila* is coloured red.

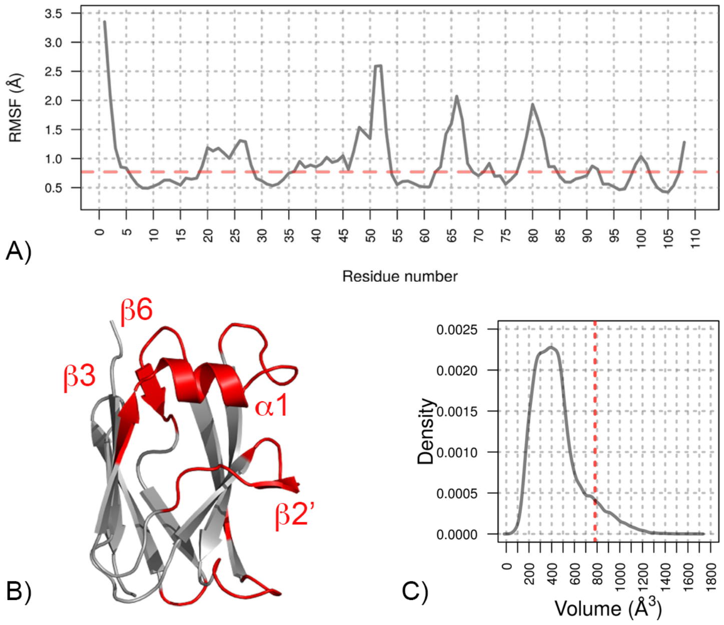

Supplementary Figure 14: NttC MD analysis. (A) Root mean square fluctuation (RMSF) analysis of the NttC ensemble. Median RMSF is shown as a red dashed line. (B) NttC X-ray structure; residues 19-28, 36-53, 63-68, 71, 72, 78-84, 91, 92, and 99-101 with RMSF >0.77 are highlighted in red. (C) Distribution of the cavity volume from NttC MD ensemble. From each structure, the cavity with highest druggability score was retained for analysis. The red dashed line represents the volume of the measured cavity in the NttC X-ray structure.

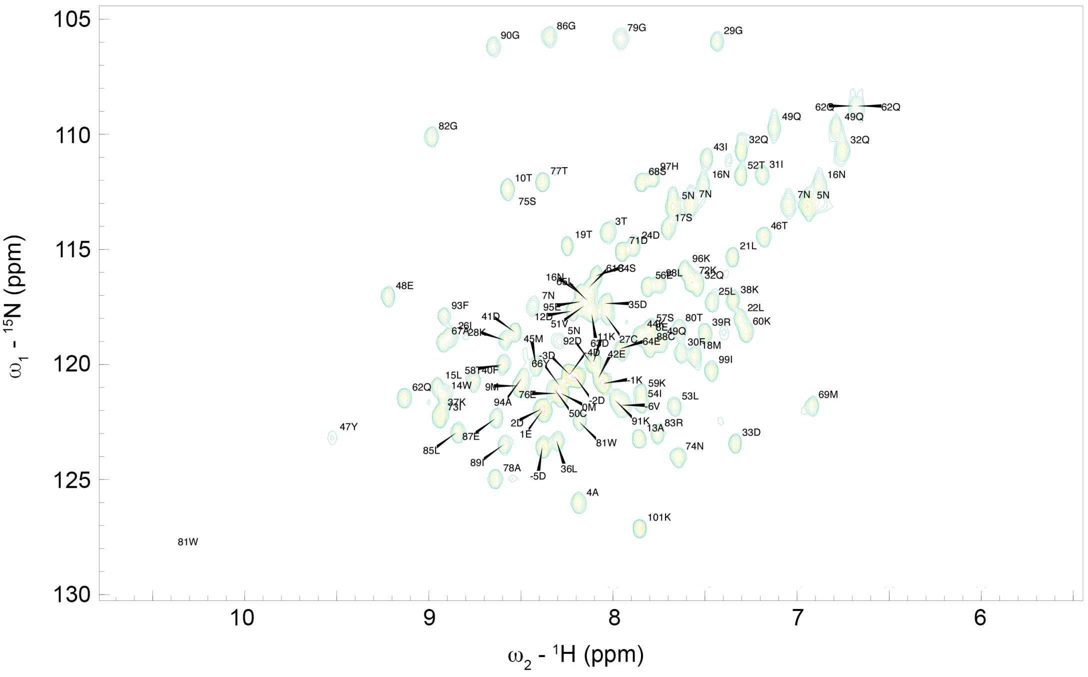

Supplementary Figure 15: 2D $^{1}H^{15}N$ HSQC NMR spectrum of ^{15}N labelled NttA. Backbone and amide side chain assignments are annotated.

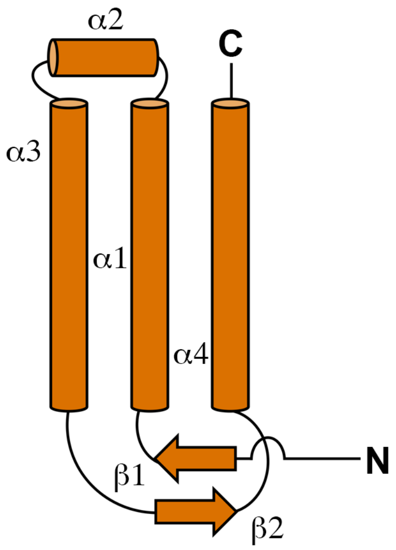

Supplementary Figure 16: Topology of NttA. Schematic representation of NttA with secondary structure coloured orange and loops in black. α-helices are labelled 1-4 and β-sheets as 1–2.

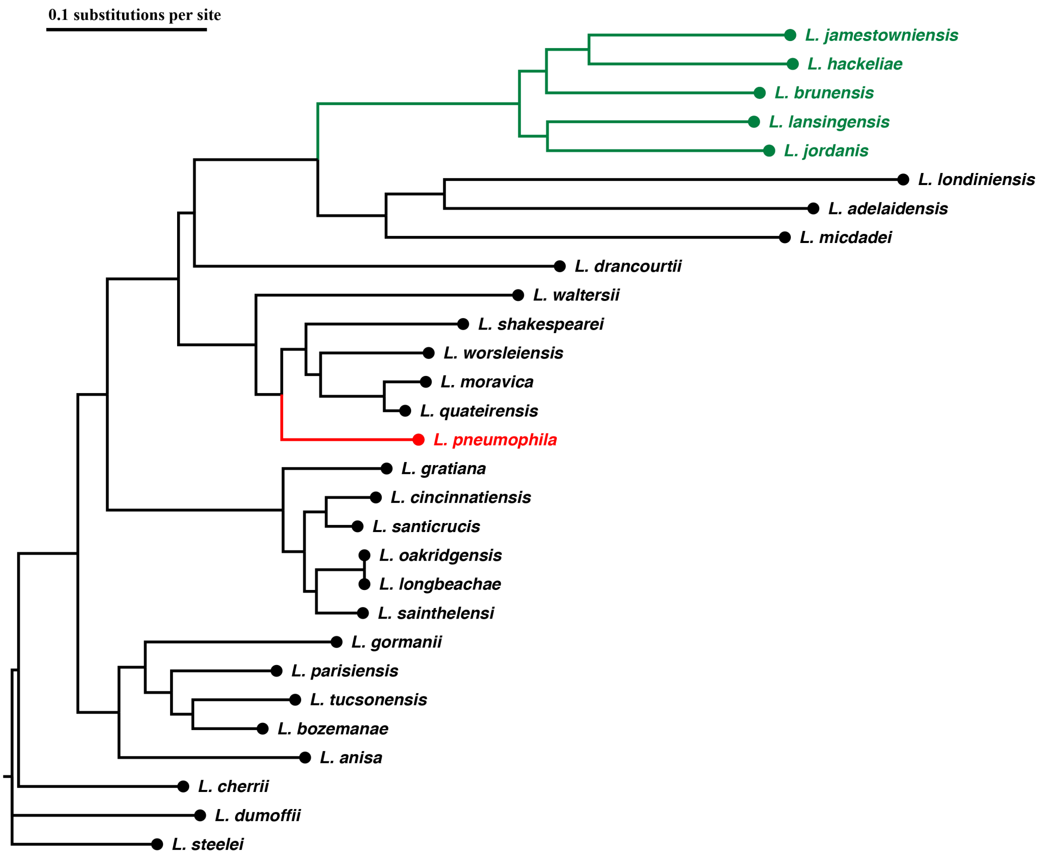

Supplementary Figure 17: Phylogenetic tree of NttA-like proteins. Scale bar indicates the number of amino acid substitutions per site. *L. pneumophila* is coloured red while *Legionella* species that contain a NttA tandem repeat are coloured green.

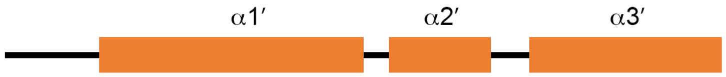


```
                                                   *                                *          *
L. jamestowniensis  ASPT-EMTKDDWLGKLKSVAPTVICQGFFEDASLKNRMEELKIDNAKCVSLIPASFDKCQ  59
L. hackeliae        ASPT-EMTKDDWLGKLKEVAPTVICQGFFEDASLKKRMEELKIDNTKCVSLIPASFEKCQ  59
L. brunensis        -APT-EMTKDDWLGKLKAVAPSVICQGFFEDASLKKRMEELKIDNDKCISLIPASFDKCQ  58
L. lansingensis     TSPT-EMTKDDWLAKLKEVAPSVICKGFFEEASLKQRMDDLKIDNAKCTSLIPDSFEKCQ  59
L. jordanis         SSPTMEMTKDDWLSKLKDVAPTVICKGFLEEPSLKKRMDELKIDNEKCMSLIPASFDKCQ  60
L. drancourtii      ------------------------------------------------------------  0
L. waltersii        ------------------------------------------------------------  0
L. shakespearei     ------------------------------------------------------------  0
L. pneumophila      ------------------------------------------------------------  0
L. worsleiensis     ------------------------------------------------------------  0
L. moravica         ------------------------------------------------------------  0
L. quateirensis     ------------------------------------------------------------  0
L. gratiana         ------------------------------------------------------------  0
L. cincinnatiensis  ------------------------------------------------------------  0
L. santicrucis      ------------------------------------------------------------  0
L. oakridgensis     ------------------------------------------------------------  0
L. longbeachae      ------------------------------------------------------------  0
L. sainthelensi     ------------------------------------------------------------  0
L. cherrii          ------------------------------------------------------------  0
L. dumoffii         ------------------------------------------------------------  0
L. steelei          ------------------------------------------------------------  0
L. gormanii         ------------------------------------------------------------  0
L. anisa            ------------------------------------------------------------  0
L. parisiensis      ------------------------------------------------------------  0
L. tucsonensis      ------------------------------------------------------------  0
L. bozemanae        ------------------------------------------------------------  0
L. londiniensis     ------------------------------------------------------------  0
L. adelaidensis     ------------------------------------------------------------  0
L. micdadei         ------------------------------------------------------------  0
R. bacterium        ------------------------------------------------------------  0
D. bacterium        ------------------------------------------------------------  0
W. bacterium        ------------------------------------------------------------  0
```

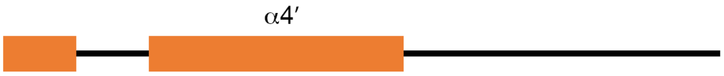


```
                                           *
L. jamestowniensis  TQYYSSLPATINKESASKWGHTIGECIGTDFATKYLVGTPPGSPDSS------------  106
L. hackeliae        TQYYSSLPSSINKESASKWGHTIGECIGTDFATKYLVATPTPGSSSSST----------  108
L. brunensis        TQYYSGLPATMNKDSAAKWGHTIGECIGTDFATKYLVSNPQASTSSSDQTTSSSTPSST  118
L. lansingensis     KQYYSNLPAMMNKESASKWGHTLGECIGTDFATKYLVSASSPSTA--------------  104
L. jordanis         KQYYSSLPANMNAQSAATWGHTLGECIGTDFATKYLVPNSQSNAP--------------  105
L. drancourtii      -----------------------------------------------------------  0
L. waltersii        -----------------------------------------------------------  0
L. shakespearei     -----------------------------------------------------------  0
L. pneumophila      -----------------------------------------------------------  0
L. worsleiensis     -----------------------------------------------------------  0
L. moravica         -----------------------------------------------------------  0
L. quateirensis     -----------------------------------------------------------  0
L. gratiana         -----------------------------------------------------------  0
L. cincinnatiensis  -----------------------------------------------------------  0
L. santicrucis      -----------------------------------------------------------  0
L. oakridgensis     -----------------------------------------------------------  0
L. longbeachae      -----------------------------------------------------------  0
L. sainthelensi     -----------------------------------------------------------  0
L. cherrii          -----------------------------------------------EDTSKST-----  7
L. dumoffii         -----------------------------------------------------------  0
L. steelei          -----------------------------------------------EDK---------  3
L. gormanii         -----------------------------------------------EDTS--------  4
L. anisa            -----------------------------------------------EDKA--------  4
L. parisiensis      -----------------------------------------------EDKT--------  4
L. tucsonensis      -----------------------------------------------------------  0
L. bozemanae        -----------------------------------------------EDKA--------  4
L. londiniensis     -----------------------------------------------------------  0
L. adelaidensis     -----------------------------------------------------------  0
L. micdadei         -----------------------------------------------------------  0
R. bacterium        -----------------------------------------------------------  0
D. bacterium        -----------------------------------------------------------  0
W. bacterium        -----------------------------------------------------------  0
```

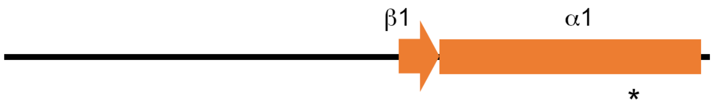


```
                                                                                     *
L. jamestowniensis    -------------------------ASNTTSSSANMSKETWFSQLKSLAPTMICQGFFDD   141
L. hackeliae          -------------------------DSSATGQPSEMPKDKWFAQLKSLAPSMICQGFFED   143
L. brunensis          SPSTPSTSPSTPSTSPSSPISSSTPATSSDSQSTDISRDQWLAQLKTLAPAMICQGFFGD   178
L. lansingensis       ---------------------------SSSTQSVDMPKDKWLEQVKALAPTMICNGFFED   137
L. jordanis           ---------------------------GSSSSSGDIPKDQWLSQLKSLAPTMICNGFFED   138
L. drancourtii        ---CANTTA-PSTTATPAAAPITAPATAPSDASFEMSKATWLTTIVPMLPSLICKGFMGD   56
L. waltersii          --------------------------EDTSASSNEMAKDAWLNLMMPMLPDLICKGFMQD   34
L. shakespearei       --------------------------EAAANNPAEMPKDSWLSSMTPLLPELICKGFIQD   34
L. pneumophila        ---------------------------EDTANPNEMTKDAWLNSMTPLLPDLICKGFIQD   33
L. worsleiensis       ---------------------------EESPSPNEMPKDNWLSSMTPLLPDLICKGFLQD   33
L. moravica           ---------------------------EDTSSPNEMPKDSWLSSMAPLLPDLICKGFIQD   33
L. quateirensis       ---------------------------EDTSSPTEMPKDSWLSSMTPLLPDLICKGFIQD   33
L. gratiana           -----------DSPSTASTTSTDTTSTSTSSTSPSLSKDEWFKSITPLLPELICKGFEND   49
L. cincinnatiensis    -----------DSPSTASTTSTDTTSTSTSSTSPSITKDEWFKSITPLLPDLICKGFEND   49
L. santicrucis        -----------DSPSTASTTNTDTTGTSTSSTSTSITKDEWFKSITPLLPDLICKGFEND   49
L. oakridgensis       -----------DSPSTASTTSTDTTGTSSSSTSPSITKDEWFKSITPLLPDLICKGFEND   49
L. longbeachae        -----------DSPSTASTTSTDTTGTSSSSTSPSITKDEWFKSITPLLPDLICKGFEND   49
L. sainthelensi       -----------DSPSTASTTSTETTGTSASSTSTSITKDEWFKSITPLLPDLICKGFEND   49
L. cherrii            --STGTTTGTGTSTSTSTSTTTNTNGAASSDSSMEISKDSWLKSVTPLLPDLICKGFEND   65
L. dumoffii           -------------------EEKSTSTGASTDNALVVSKEQWLKAVTPLLPDLICKGFEND   41
L. steelei            ----------STSTSTTSPNAKSTSNTGAAPGSSEISKDSWLKSVTPLLPDLICKGFEND   53
L. gormanii           ----------GGISSSGVSSNNNASSNATSNNPVDISKDSWLKSVTPLLPDLICKGFEND   54
L. anisa              ----------DSSTSS--STGS-----STDSATDSISKESWLKSVTPLLPDLICKGFQND   47
L. parisiensis        ----------ASSTSS--ST---GSSTDTSNSTINISKDSWLKSVTPLLPDLICKGFQND   49
L. tucsonensis        ------------------EDKTDSSTDTSSNSTIEISKDSWLKSVTPLLPNLICKGFEND   42
L. bozemanae          ----------GGSTSS--STGTSSSTDSSSNSTIDISKDSWLKSVTPLLPDLICKGFEND   52
L. londiniensis       ----------------------------ATNAKMEMSKDEWLSKIKEIVPEPICKGFMQD   32
L. adelaidensis       --------ATDTAPKSDGSQPTGTSAGASNRTVTQVPKDEWLKKIKEIVSEPICKGFVED   52
L. micdadei           ---------------------------------DGLSKDDWINSLKDLAPAAVCKSFVQD   27
R. bacterium          ---------------------------------QGLDKASWIKGFRPVMTDMFCKN----   23
D. bacterium          ---------------------------------ETLSKKEWGDAMKSGLPVLLCKR---D   24
W. bacterium          --------------------------------QDSISREAWSAYMQDALPEFLCHE---E   25
```

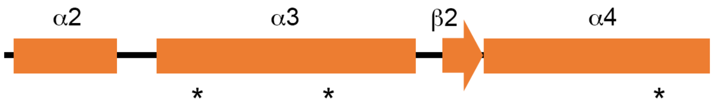


```
                             *            *                               *
L. jamestowniensis    ASIKKKLDDRNIDNAKCITLIAPSFDKCQSEYYTNLPSSID-SEAANTWGRKIGECIGTD   200
L. hackeliae          ASLKKKLEDRNIDNTKCVSLISPSFEKCQTEFYSSLPATID-NDTANTWGRKIGECIGTD   202
L. brunensis          PSLKKKLEDRSIDNTKCVNLIPASFEKCQNELYSNLPTTLN-SDSANTWGHKIGECIGTD   237
L. lansingensis       ESIKKKLQGRNIDNAKCMTLIPESFDKCQTQYYSSLPSTLN-VETANTWGHKIGECIGTD   196
L. jordanis           ESIKKKLQDRGIDNAKCVTLIPASFDKCQNEFYSNLPGTLN-AENANTWGHKMGECIGTD   197
L. drancourtii        PDLKQRLDAIKMSYEQCVSAIPESVSKCQNQLYASIPEQIS-STDAATWGKKFGECIGKD   115
L. waltersii          AELKQRFDELKLTFEQCVTLIPESSNKCKDELYASIPDKVN-SESASTWGKSLGECIGKD   93
L. shakespearei       ADLKKRFDEIKMTYEQCVTLIPESTTKCQNELYANIPATIN-AESAATWGRSLGECIGRD   93
L. pneumophila        PDLKKRFDEIKMTYEQCVTLIPESTKKCQDELYASMPDKIN-SETAGTWGRSLGECIGKD   92
L. worsleiensis       ADLKKRFDEIKMTYEQCVTLIPASTSKCQNELYAKMPDKIN-SESAGTWGRSLGECIGRD   92
L. moravica           ADLKKRFDEIKMTYEQCVTLIPESTNKCQNELYGSMPDKIN-SESAAVWGRSLGECIGKD   92
L. quateirensis       ADLKKRFDEIKMTYEQCVTLIPESTNKCQNELYGSMPDKIN-SESAATWGRSLGECIGKD   92
L. gratiana           AQLKKRLDDIKMTHEQCLAAIPESVSKCQQQIYANVPDKLT-DESAATWGKNLGECIGKD   108
L. cincinnatiensis    AQLKKRLDDIKMTHEQCLTAIPESVSKCQQELYTKIPDKLN-DETTATWGKTLGECIGKD   108
L. santicrucis        AQLKKRLDDIKMTHEQCLTAIPESVSKCQQELYSKIPDKLN-DETAATWGKTLGECIGKD   108
L. oakridgensis       AQLKKRLDDIKMTHEQCLSAIPESVSKCQQELYAKIPDKLT-DETAANWGKALGECIGKD   108
L. longbeachae        AQLKKRLDDIKMTHEQCLSAIPESVSKCQQELYAKIPDKLT-DETAANWGKALGECIGKD   108
L. sainthelensi       AQLKKRLDDIKMTHEQCLAAIPESVSKCQQDLYAKIPDKLT-DETAATWGKSLGECIGKD   108
L. cherrii            PQLKKRLDDIKMNYDKCIAAIPESVSKCQQQLYGSIPDKIN-NDSAAVWGKSLGECIGKD   124
L. dumoffii           PQLKKRLDDIKMNYEQCVAKIPDSISKCQQQLYGSIPDKID-NDSAAVWGKSLGECIGKD   100
L. steelei            PQLKKRLDDIKMSYDQCVAAIPDSVSKCQQQLYGSIPDKIN-DDSAAVWGKSLGECIGKD   112
L. gormanii           PQLKKRLDEIKMTYEQCATKIPDSVSKCQQELYANIPDKIN-SENAGIWGKALGECIGKD   113
L. anisa              PQLKKRLDEIKMTFEQCVAKIPDSVSKCQQDIYASIPEKIT-NDNAGVWGKALGECIGKD   106
L. parisiensis        PQLKKRLDDIKMTYDQCVTKIPDSVSKCQQELYANIPDKIN-TDNAGVWGKALGECIGKD   108
L. tucsonensis        SQLKKRLDEIKMTYDQCVVKIPDSVSKCQQDLYANIPDKIN-TDNAGVWGKALGECIGKD   101
L. bozemanae          PQLKKRLDDIKMTYDQCVSKIPDSVSKCQQELYANIPDKIN-TENAGVWGKALGECIGKD   111
L. londiniensis       ETIASRLKARNIDLQKCIELIPGIADKCSKKFYDRLPATIT-QENVSKWGHSIGECIGAE   91
L. adelaidensis       ASISERLKEQKISYEKCVTLIPPIAEKCEKKYYDSIPAMIT-EDNASKWGRMIGECIGAD   111
L. micdadei           NDLSKRLKEANISYDKCLTLIPTSFDKCQAKYYSQIPDKIT-KKEAEKWGNNIGMCIGGD   86
R. bacterium          PLI---QKCFKVSQQKCLEVIGQTTDSCLRKFDSSLPAFFQMPADGRKWGRDVGQCAGDE   80
D. bacterium          EYF---RDCFKISQEECEDIIASATRVCFKQIETQIPSKIVQPRDGEKWGRKIGECVGVS   81
W. bacterium          SYF---VECFEVDIEQCKRSLSALARSCLESLAEHIPDPIRSAQEGATAGQMIGSCAGSR   82
```

```
L. jamestowniensis      FAKKYLVSAPSADT--TSATTTTSPSRT---SAPVSTT------PNQ   236
L. hackeliae            FAKKYLVNAPASTS--TPADVETPSSTTSTTTVPATTAPSTTTAPGQ   247
L. brunensis            FAKKYLLPTATPAT--STSSQ--------------------------   256
L. lansingensis         FAKKYLVPAETTTN--GQ-----------------------------   212
L. jordanis             FAKQYLVSSEAKSN--APQKSSSPSEASSKDNQ--------------   228
L. drancourtii          FAMKYLLPK--------------------------------------   124
L. waltersii            FAEKHLIPKNE------------------------------------   104
L. shakespearei         FAEKYLVPKQ-------------------------------------   103
L. pneumophila          FAEKHLIPK--------------------------------------   101
L. worsleiensis         FAEKYLVPKN-------------------------------------   102
L. moravica             FAEKYLVPKN-------------------------------------   102
L. quateirensis         FAEKYLVPKS-------------------------------------   102
L. gratiana             FAIKYLIPKS-------------------------------------   118
L. cincinnatiensis      FAIKYLIPKN-------------------------------------   118
L. santicrucis          FAIKYLIPKS-------------------------------------   118
L. oakridgensis         FAIKYLIPKS-------------------------------------   118
L. longbeachae          FAIKYLIPKS-------------------------------------   118
L. sainthelensi         FAIKYLIPKS-------------------------------------   118
L. cherrii              FALKYLIPKS-------------------------------------   134
L. dumoffii             FAIKYLIPQS-------------------------------------   110
L. steelei              FAIKYLIPKS-------------------------------------   122
L. gormanii             FAMKYLIPKSQ------------------------------------   124
L. anisa                FAIKYLVPKS-------------------------------------   116
L. parisiensis          FAIKHLIPKN-------------------------------------   118
L. tucsonensis          FAIKYLIPKS-------------------------------------   111
L. bozemanae            FAIKYLIPKS-------------------------------------   121
L. londiniensis         FVINYVFSDTEEKK--DTTADGE-QDAKTNSEMKKET--GN------   127
L. adelaidensis         FAVNYLYPGSGTSA--SADSTSSKSDNSTNGSSKSSN--GASQKSGQ   154
L. micdadei             FTVNYLLVPK-------------------------------------   96
R. bacterium            FESVLIADKVKGGECGP------------------------------   97
D. bacterium            AETTLTDDKISNKKCNDPNAWE-------------------------   103
W. bacterium            YDLVNLALKISTEQCNDPMYWAGRQ----------------------   107
```

Supplementary Figure 18: Sequence alignment of *L. pneumophila* NttA and other NttA-like proteins. Secondary structure elements of NttA are shown above a (rectangle: α-helix; arrow: β-strand). Amino acid identities and similar residues are indicated by background shading in cyan and yellow, respectively. Disulfide forming cysteine positions are indicated by an asterisk. *L. pneumophila* is coloured red while *Legionella* species that contain a NttA tandem repeat are coloured green.

```
                                                     *                      *
L. pneumophila          EDTANPNEMTKDAWLNSMTPLLPDLICKGFIQDPDLKKRFDEIKMTYEQCVTLIPESTKK  60
L. jordanis             --SSPTMEMTKDDWLSKLKDVAPTVICKGFLEEPSLKKRMDELKIDNEKCMSLIPASFDK  58
L. brunensis            ----APTEMTKDDWLGKLKAVAPSVICQGFFEDASLKKRMEELKIDNDKCISLIPASFDK  56
L. jamestowniensis      ---ASPTEMTKDDWLGKLKSVAPTVICQGFFEDASLKNRMEELKIDNAKCVSLIPASFDK  57
L. hackeliae            ---ASPTEMTKDDWLGKLKEVAPTVICQGFFEDASLKKRMEELKIDNTKCVSLIPASFEK  57

                        *                         *
L. pneumophila          CQDELYASMPDKINSETAGTWGRSLGECIGKDFAEKHLIPK- 101
L. jordanis             CQKQYYSSLPANMNAQSAATWGHTLGECIGTDFATKYLVPNS 100
L. brunensis            CQTQYYSGLPATMNKDSAAKWGHTIGECIGTDFATKYLVSNP 98
L. jamestowniensis      CQTQYYSSLPATINKESASKWGHTIGECIGTDFATKYLVGTP 99
L. hackeliae            CQTQYYSSLPSSINKESASKWGHTIGECIGTDFATKYLVATP 99
```

Supplementary Figure 19: Sequence alignment of *L. pneumophila* NttA with the N-terminal sequence of tandem NttA proteins. Amino acid identities and similar residues are indicated by background shading in cyan and yellow, respectively. Disulfide forming cysteine positions are indicated by an asterisk. *L. pneumophila* is coloured red while *Legionella* species that contain a NttA tandem repeat are coloured green.

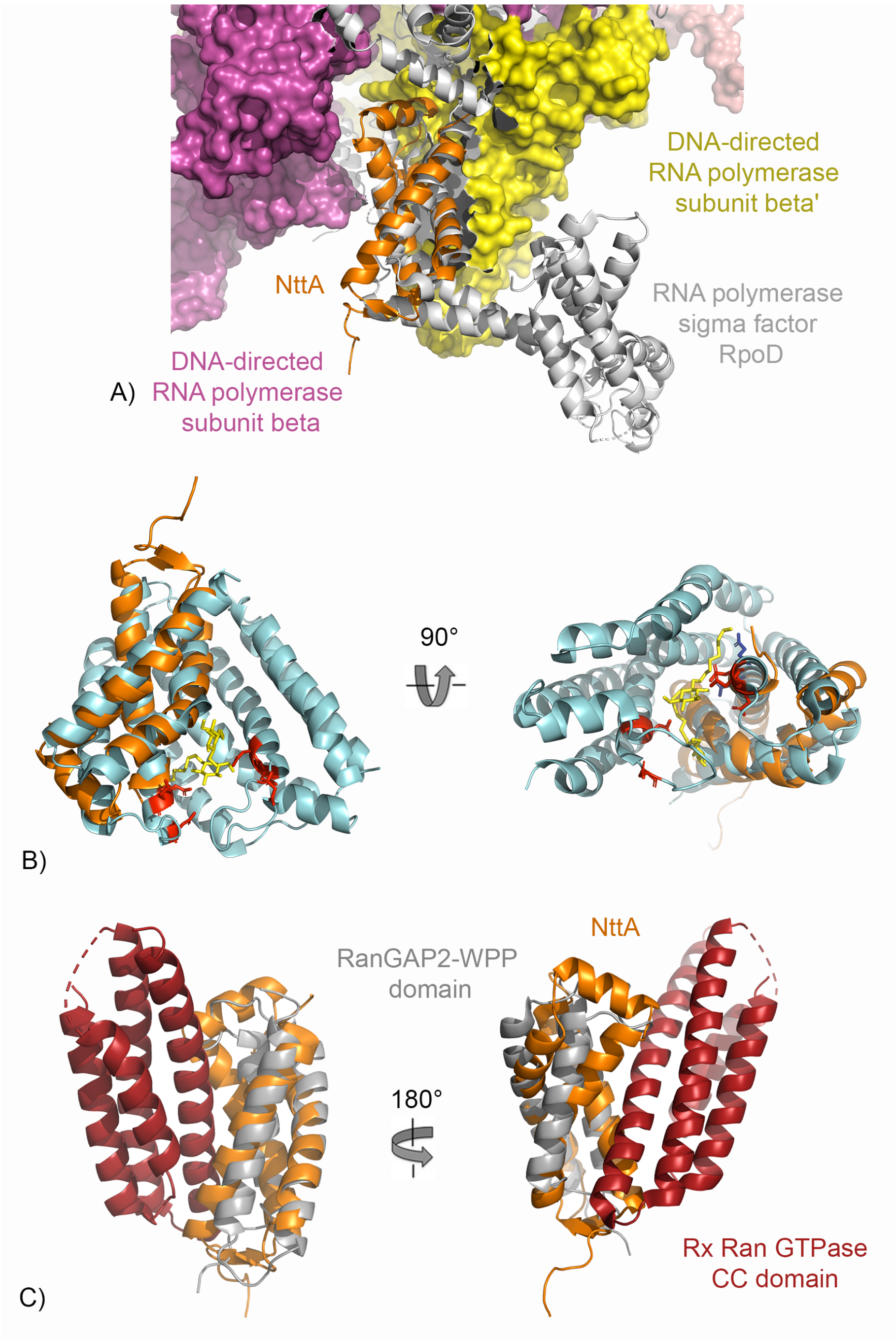

Supplementary Figure 20: Structural homology with NttA. (A) Superposition of NttA (orange) onto RpoD (grey) within the RNA polymerase complex (PDB ID code 4LK1). (B) Superposition of NttA (orange) onto MoeN5 (cyan) bound to β-nonylglucoside (yellow; PDB ID code 5B0L). Red sticks in MoeN5 represent Asp residues from two DDxD motifs. Blue sticks in NttA represent adjacent acidic residues in NttA that may have a similar role. (C) Superposition of NttA (orange) onto RanGAP2 (grey) from the RanGAP2/Rx complex (PDB ID code 4M70).

1.2 Supplementary Tables

Supplementary Table 1: Primers used in this study

Primer	Sequence (5' to 3')
JS3	TGACCCAAGCCATTGTGTTC
SB34	AAAAAACCCGGGTTTTCTACATCATCACAGC
JS4	AAAAAACCCGGGAAAATCTCCCCTAATCTGT
SB35	GCGCGTTACATCCGTTGTTT
RW1	AAAGGTACCTTGCTGAAGAGGCGTGTCTAAGTA
RW2	GAAGCAGCTCCAGCCTACACAAACAAACTCCTTTTAAATTAATTCAGAA
RW3	TAAGGAGGATATTCATATGCTAATGAAATCAGCAGAAACCG
RW4	GCCAGTGTTAATTTTGGAAAGGAC
RW5	TTCTGAATTAATTTAAAAGGAGTTTGTTTGTGTAGGCTGGAGCTGCTTC
RW6	CGGTTTCTGCTGATTTCATTAGCATATGAATATCCTCCTTA
JG21	GACGACGACAAGATGGAGGACACAGCAAATCCTAATGAG
JG22	GAGGAGAAGCCCGGTTATTTGGGAATTAGATGTTTTTCAGCAAAATCTTTTC
JG23	GACGACGACAAGATGGCTCCAGCTTATTTAACAACCC
JG24	GAGGAGAAGCCCGGTTAGTTTTTAGTAATTTCAGCCTCTCCAGG

SmaI sites are underlined
